# Evolution of T cell receptor beta loci in salmonids

**DOI:** 10.3389/fimmu.2023.1238321

**Published:** 2023-08-15

**Authors:** Pierre Boudinot, Samuel Novas, Luc Jouneau, Stanislas Mondot, Marie-Paule Lefranc, Unni Grimholt, Susana Magadán

**Affiliations:** ^1^ Université Paris-Saclay, INRAE, UVSQ, VIM, Jouy-en-Josas, France; ^2^ Immunology Laboratory, Research Center for Nanomaterials and Biomedicine (CINBIO), Universidade de Vigo, Vigo, Spain; ^3^ Université Paris-Saclay, INRAE, AgroParisTech, Micalis Institute, Jouy-en-Josas, France; ^4^ IMGT^®^, The International ImMunoGeneTics Information System® (IMGT), Laboratoire d´ImmunoGénétique Moléculaire (LIGM), Institut de Génétique Humaine (IGH), Centre National de la Recherche Scientifique (CNRS), University of Montpellier, Montpellier, France; ^5^ Fish Health Research Section, Norwegian Veterinary Institute, Oslo, Norway

**Keywords:** T cell receptor, salmonid fish, repertoire, TRB locus, adaptive immunity, evolution, rainbow trout

## Abstract

T-cell mediated immunity relies on a vast array of antigen specific T cell receptors (TR). Characterizing the structure of TR loci is essential to study the diversity and composition of T cell responses in vertebrate species. The lack of good-quality genome assemblies, and the difficulty to perform a reliably mapping of multiple highly similar TR sequences, have hindered the study of these loci in non-model organisms. High-quality genome assemblies are now available for the two main genera of Salmonids, *Salmo* and *Oncorhynchus*. We present here a full description and annotation of the TRB loci located on chromosomes 19 and 25 of rainbow trout (*Oncorhynchus mykiss*). To get insight about variations of the structure and composition of TRB locus across salmonids, we compared rainbow trout TRB loci with other salmonid species and confirmed that the basic structure of salmonid TRB locus is a double set of two TRBV-D-J-C loci in opposite orientation on two different chromosomes. Our data shed light on the evolution of TRB loci in Salmonids after their whole genome duplication (WGD). We established a coherent nomenclature of salmonid TRB loci based on comprehensive annotation. Our work provides a fundamental basis for monitoring salmonid T cell responses by TRB repertoire sequencing.

## Introduction

Vertebrate adaptive immunity relies on the clonal expression of somatically diversified antigen receptors on lymphocytes. In jawed vertebrates, the adaptive immune components include B and T lymphocytes, which express antigen-specific receptors, the immunoglobulins (IG) or antibodies and T cell receptors (TR), respectively (1–6). T lymphocytes can be classified into two main lineages based on the TR they express, either an alpha/beta or a gamma/delta heterodimer (6, 7). Conventional T helper and T cytotoxic cells express TR alpha/beta receptors, which recognize peptide antigens presented in the context of Major Histocompatibility Complex (MHC) proteins (7, 8). Each TR chain contains a variable (V) domain, a constant (C) domain, a connecting region (CO), a transmembrane region (TM) and a short cytoplasmic tail (CY) (6). Genes encoding the variable domain, called variable (V), diversity (D) for the beta and delta chains, and joining (J) undergo somatic rearrangement mediated by the recombinases encoded by recombination activating genes 1 and 2 (*rag1/2*) and other enzymes, during the T lymphocyte differentiation. These V-(D)-J rearrangements are imprecise and generate a large sequence diversity required for the antigen-specific recognition by T cells. Characterizing the structure of TR loci is essential to get insight about the diversity and composition of T cell responses in vertebrate species.

The analysis of TRB loci in different mammals has confirmed a common genomic structure (9), with different numbers of TRBV genes positioned upstream of tandem-aligned TRBD-J-C clusters, each composed of a single D (TRBD) gene, several J (TRBJ) genes, and one constant (TRBC) gene. Following the last TRBD-J-C cluster, a single TRBV is found in inverted transcriptional orientation, which rearranges by inversion (6). While this general structure is well conserved, the number of TRBV genes and TRBD-J-C clusters may vary between species (9), likely due to partial duplications of the locus during evolution. Comparing TRB loci and expressed T cell repertoires between mammals and ectothermic vertebrates like bony fish provides important insights into the evolutionary history of the adaptive immune system. However, studies aiming to annotate antigen receptor gene loci in non-model organisms have been hampered by the lack of high-quality genome assemblies. The recent release of high-quality genome assemblies for the main genera of Salmonids (family Salmonidae) provides the opportunity to compare TRB loci across these economically important species. While a whole genome duplication occurred in early teleosts (10), the ancestors of salmonids underwent an additional round of genome duplication, known as the salmonid-specific whole-genome duplication (ssWGD), approximately 80-100 million years ago (MYA) (11–13). The ssWGD contributed to genomic and phenotypic innovation as well as speciation, providing salmonids with a unique opportunity to acquire new genes. We previously reported that salmonid IGH are encoded by two independent rearranging loci located on different chromosomes, resulting from the ssWGD (14). In contrast, only one copy of the TRAD locus has been retained in both *Salmo* and *Oncorhynchus* species (15).

In the 1990s, TRB chain cDNAs were cloned in rainbow trout (*Oncorhynchus mykiss*) and later in Atlantic salmon (*Salmo salar*) (16–19). A partial description of the organization of the TRB locus was reported from rainbow trout (20), in which a TRBD gene, 10 TRBJ genes and the 5’-end of the first TRBC exon were identified in a 5.5 kb genomic segment of a trout TRB locus. Further analysis of rainbow trout TRB transcripts expressed in spleen allowed the identification of 10 TRBV subgroups, consisting of sequences where each subgroup share more than 75% nucleotide identity (21). The recent description of TRB loci in Atlantic salmon uncovered an intriguing configuration, with two regions on chromosomes (chr.) 1 and 9 (22), each composed of two V_(n)_-D_(1)_-J_(k)_-C_(1)_ clusters in inverted orientation, defining four TRB loci. A total of 119 TRBV genes were annotated in the Atlantic salmon genome, most of them clustering with the 10 subgroups previously identified in rainbow trout (21). The genomic organization of Atlantic salmon TRB loci differs from that found in zebrafish (*Danio rerio*), channel catfish (*Ictalurus punctatus*) and mammals, in which a variable number of TRBV genes are followed by two or three TRBD-J-C clusters in tandem (23–26). The phylogenetic analysis also defined three new subgroups consisting of 24, 1 and 4 TRBV genes. Although the total number of Atlantic salmon TRBV genes is higher than in zebrafish (51 TRBV genes) and catfish (102 TRBV genes), these segments are less diverse and constitute fewer subgroups (13 in Atlantic Salmon versus 36 in zebrafish and 30 in catfish) (23, 24). The analysis of catfish transcriptomes and cDNA sequences revealed that at least 17 out of 30 TRBV subgroups are expressed, and most of those not detected corresponded to single-member subgroups, which may have been easily missed (23). In-depth examination of zebrafish and Atlantic salmon TRB repertoire using high-throughput sequencing, indicated that in both species most of TRBV subgroups are indeed expressed, even those with only one member (22, 27).

High-quality genome assemblies are available for the two main genera of Salmonids, *Salmo* and *Oncorhynchus*. To get insights into variations of the structure and composition of TRB locus across salmonids, we undertook a full description and annotation of the TRB loci in rainbow trout (*Oncorhynchus mykiss*). A comparison with other salmonid species confirmed that the basic structure of salmonid TRB locus is a double set of two TRBV-D-J-C loci duplicated in opposite orientation on two different chromosomes.

The growing interest for the dynamics of adaptive immune responses in farmed salmonids has resulted in recent efforts to develop deep sequencing protocols to monitor B cell and T cell repertoires during immune responses (15, 22). Establishing a comprehensive and coherent salmonid TRB loci description and nomenclature is a fundamental prerequisite for accurate annotation of repertoire sequencing datasets. Although this task is especially challenging due to the presence of large duplicated loci, it is therefore worthwhile and it also shed light on the evolution of antigen specific receptors through WGD.

## Materials and methods

### Annotation of TRB loci

Rainbow trout Arlee (USDA_OmykA_1.1; GenBank GCA_013265735.3, isolate Arlee) and Swanson (Omyk_2.0; GenBank GCA_025558465.1, isolate Swanson DH line) genome assemblies were accessed through the NCBI website (https://www.ncbi.nlm.nih.gov/). To identify the chromosomes that contain the TRB loci, previously published salmonid TRBC sequences (20, 22) were used for BLASTn and tBLASTn searches. The regions encoding for TRBC were located on chr. 19 and 25, in rainbow trout Arlee [Chr19: CM023237.2, NC_048583.1, 67237266 base pairs (bp) and Chr25: CM023243.3, NC_048589.1, 47542702 bp] and Swanson (Chr19: CM046588.1, 62815373 bp and Chr25: CM046594.1, 92872484 bp). These chromosomes were selected for in depth analysis. TR genes were searched using the BLAST tool at the Galaxy website (https://usegalaxy.org) and visual analysis using SnapGene software (from Insightful Science; available at snapgene.com). Previously published TRB constant sequences from Atlantic salmon (22) were again used as queries to identify the chromosomal regions containing TRBC genes, intron splice signals were identified for all sequences and were used to determine the limits of the TRBC coding exons. The annotation and functionality of TRB genes were established according to IMGT rules and standards (25). To annotate TR variable V, joining J, and diversity D genes, the recombination signal (RS) sequences were identified, respectively V-RS, J-RS, and 5’D-RS and 3’D-RS. Splice signals were used to determine the limits of the coding nucleotide sequences for the V (L-PART1 donor splice and L-PART2 acceptor splice) and the J (J-REGION donor splice). *In silico* analysis of gene function considered the following parameters: a) presence of appropriate splice sites, b) presence of RS sequence compatible with effective rearrangement, c) open reading frames which included conserved cysteine (CYS) and tryptophan (TRP) codons at positions 23 (1st-CYS), 41 (CONSERVED-TRP) and 104 (2nd-CYS) of TRBV genes (IMGT unique numbering system) (28). Briefly, a germline entity (TRBV, TRBD or TRBJ gene) was considered as a functional gene (F) if the coding region has an open reading frame, no defect in splicing site or in RS, and presents key conserved amino acids; as open reading frame (ORF) if the coding region has an open reading frame with defects in the splicing sites or RS sequences, and/or changes of conserved amino acids that lead to incorrect folding; or annotated as pseudogene (P) if the coding region has stop codon or frameshift mutations (28–30).

For comparative analysis we used genome assemblies from other Salmonid species that are available at NCBI and/or Ensembl. Specifically, Atlantic salmon (*Salmo salar*): GCA_905237065.2, brown trout (*Salmo trutta*): GCA_901001165.1, Coho salmon (*Oncorhynchus kisutch*): GCA_002021735.2, Chinook salmon (*Oncorhynchus tshawytscha*): GCA_002872995.1, Pink salmon (*Oncorhynchus gorbuscha*) (even and odd year): GCA_021184085.1 and GCA_017355495.1. Pink salmon lives strict two-year life cycles, where odd and even year populations do not mix. We also included Northern pike (*Esox lucius*): GCA_004634155.1, as a representative species belonging to a sister group of Salmonids, which split from the salmonid lineage prior to the fourth salmonid specific whole genome duplication.

### Rainbow trout TRBV genes

Rainbow trout TRBV genes were named based on nucleotide similarity, phylogenetic analysis, and positional information. We followed the same principles as in our recent work on salmonid IGH and TRA/TRAD loci (14, 15), using the classification and nomenclature of Atlantic salmon TRBV genes (22) as a reference, which used the 10 TRBV subgroups originally proposed in (21), and complying with the IMGT (https://www.imgt.org/) criteria (28, 31). We then mapped rainbow trout TRBV genes to the corresponding subgroups based on a threshold of 75% nucleotide identity and named them based on positional information. Thus, TRBV names are constituted as follows: first, TRB, a locus number (1 to 4) defining the associated D-J-C cluster, then the letter V with the subgroup number, which is followed by a dash and a number (N1) that denotes the gene rank in the locus, from 5´to 3´. For example, the name TRB2V13-4 denotes a gene belonging to the subgroup 13, located at the reference rank 4 within locus TRB2. So, in the TRB2 locus, there are at least three more TRBV genes belonging to subgroup 13, which are located upstream to TRB2V13-4. Salmonid TRB nomenclature proposed in this manuscript has been approved as official nomenclature by the International Union of Immunological Societies (IUIS) Nomenclature Committee (NOM), Immunoglobulins (IG), T cell receptors (TR) and major histocompatibility (MH) SubCommittee (IMGT-NC), as reported in: IUIS-NOM-IMGT-NC_Report_2022-2-0429_Salsal_TRB, for Atlantic salmon (32); and IUIS-NOM-IMGT-NC_Report_2022-3-0722_Oncmyk_TRB, for rainbow trout (33). Chromosomal coordinates given in the Atlantic salmon and rainbow trout TRB gene annotation file (**Supplementary File 1**) include the recombination signal (RS) sequence and coding regions of TRBV, TRBJ, and TRBD genes, and refer to the genomic region that contains the 3 exons of the TRBC genes. The **Supplementary File 1** includes the correspondence between the IMGT nomenclature (32) and the gene name which has been used in the previous work about Atlantic salmon TRB genes (22).

### Phylogenetic analysis

Phylogenetic analysis was performed on TRBV or TRBC sequences from rainbow trout, Atlantic salmon and other salmonids. Phylogenetic trees were constructed based on sequences aligned by ClustalW, using MEGA X (34). Trees were inferred using the Neighbor Joining method (pairwise deletion, with a JTT matrix-based model). In all phylogenetic trees the number of bootstrap replications was 1000, and the consensus tree was shown.

### Expression of TRB genes

The expression of TRBC genes was assessed in publicly available transcriptome data across from different salmonid species. Transcriptome sequencing data was downloaded from Sequence Read Archive (SRA). Specifically: *O. mykiss* (SRR11972660, SRR11972662, SRR11972726); *O. gorbutscha* (SRR9841114); *S. trutta* (SRR6666113); *O. tshawytscha* (SRR6273681, SRR3986817, SRR3986815); *O. kisutch* (SRR5333378, SRR5333374, SRR5333360). Sequences were cleaned using cutadapt and were mapped against TRBC1 and TRBC2 transcript sequences using Bowtie2 (option –local). Mapping (bam) files were filtered using samtools (35, 36), allowing for a maximum of two mismatches in sequence alignments. Numbers of successful alignments for TRBC1 and TRBC2 were then counted, and counts were transformed in RPKM values considering library sequencing depth and the length of TRBC sequences.

## Results

### Rainbow trout TRB genomic organization

**Figure 1:**
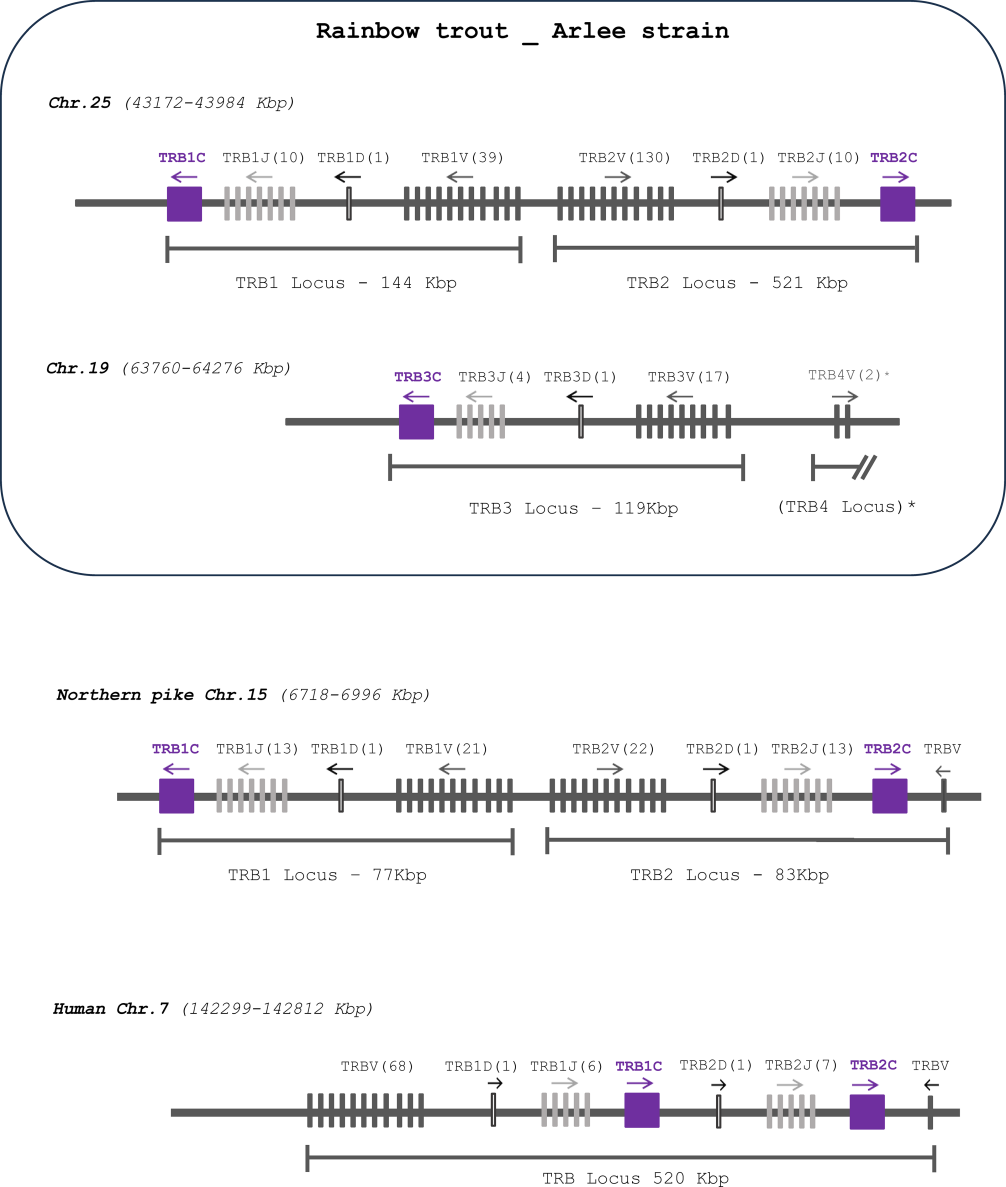
Comparative genomic organization of TRB loci. Gene organization of TRB loci in rainbow trout (*Oncorhynchus mykiss*, Arlee strain) compared to those of Northern pike (*Esox lucius*) and human (*Homo sapiens*). TRBC genes as shown as purple boxes, TRBJ and TRBV genes as light and dark grey lines, respectively; and TRBD genes as white lines. The number of corresponding genes is between brackets. The arrow indicates the transcriptional orientation. The symbols representing the genes are not to scale. See **Supplementary File 2** for exact location of each gene. * TRBV genes located approximately 390 Kbp downstream to the previous TRBV gene. TRB4 in parenthesis indicates the absence of D-J-C cluster.

Rainbow trout TRB loci were annotated from the newly released genome assembly of the Arlee clonal strain of rainbow trout (USDA_OmykA_1.1), data available in GenBank (GCA_013265735.3) as well as in Ensembl (Release 108). Rainbow trout TRB genes were identified within three TRB loci named TRB1, TRB2 and TRB3, that were located on chr. 25 (TRB1 and TRB2) and 19 (TRB3). These three TRB loci follow a similar pattern of translocon configuration, with a number of TRBV genes followed by one TRBD, several TRBJ genes, and one TRBC gene (**Figure 1**; **Supplementary File 2**). Specifically, the TRB1 locus (NC_048589.1) spans 144 Kb and contains a total of 51 genes: 39 TRBV genes (35 F, 4 P), 1 TRBD, 10 TRBJ and 1 TRBC genes. The TRB2 (NC_048589.1) locus spans 521 Kb and presents a total of 142 genes: 130 TRBV genes (113 F, 8 ORF, 9 P), 1 TRBD, 10 TRBJ and 1 TRBC genes. The TRB3 (NC_048583.1) locus is the smallest one, covering a region of 119 Kb and a total number of 23 genes, 17 TRBV genes (12 F, 4 ORF, 1 P), 1 TRBD, 4 TRBJ and 1 TRBC genes. The V-D-J-C TRB1 and TRB2 loci are in opposite transcriptional orientations (**Figure 1**; **Supplementary File 2**). The number of TRB loci present in the rainbow trout USDA_OmykA_1.1 genome assembly differs from what was recently described in Atlantic salmon (22), which has four TRB loci, two of them, TRB1 and TRB2, located on chromosome 9, and the other two, TRB3 and TRB4, on chromosome 1. The genome assembly from the Swanson clonal line of rainbow trout (Omyk_2.0 GenBank assembly [GCA_025558465.1]), as well as the Arlee rainbow trout genome assembly described in this paper, only contains three complete TRB V-D-J-C loci (two on chr. 25 and one on chr. 19); however, 2 additional TRBV genes in inverted orientation were present on chr. 19. Their localization suggests they may be the remnant of a TRB4 locus. The rest of this fourth locus might have been lost during evolution in this species, or alternatively might result from a gap in the rainbow trout genome assemblies (**Figure 1**; **Supplementary File 2**).

### Comparison of D-J-C clusters

**Figure 2:**
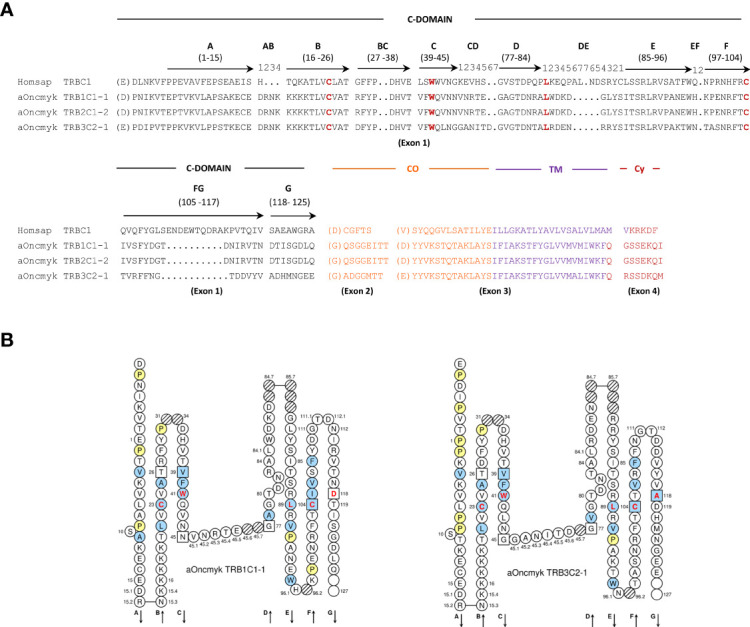
Deduced amino acid sequences from rainbow trout TRBC genes annotated in the USDA_OmykA_1.1 genome (GenBank GCA_013265735.3, isolate Arlee). **(A)** Alignment of the TRBC amino acid sequences from human (Homsap) and rainbow trout (aOncmyk). The domains and regions are indicated as follow: C-DOMAIN (constant domain), CO (connecting region), TM (transmembrane region) and CY (cytoplasmic region). The four conserved amino acids of the C-DOMAIN: 1st-CYS 23 and 2nd-CYS 104 (cysteins of the disulfide bridge), CONSERVED-TRP 41 (tryptophan, W) and the hydrophobic amino acid at position 89 are in red. Description of the C domain strands (A, B, C, D, E, F, G), turns (AB, DE, EF), loops (BC, FG), and their IMGT positions are according to the IMGT unique numbering for C-DOMAIN (29). Missing positions 32 and 33 in the BC loop are not shown. The amino acids between parentheses at the beginning of EX1, EX2 and EX3 corresponds to the first codon resulting from a splicing frame 1. The splicing between EX3 and EX4 is a splicing frame 0. **(B)** IMGT Collier de Perles of the rainbow trout TRB1C1-1 and TRB3C2-1 genes as derived from the alignment with the human TRBC proteins.

The three rainbow trout TRBC genes contain four exons that encode the characteristic constant C domain (29, 31), with the four conserved amino acids, the two Cys of the disulfide bridge at IMGT positions 23 and 104, Trp at position 41 and the hydrophobic amino acid at position 89 (29, 31), the connecting region (CO), the transmembrane region (TM), and the cytoplasmic region (CY) (**Figure 2**). Each TRBC gene encodes a sequence of 168 (TRB3C), 169 (TRB1C) and 170 (TRB2C) amino acids. TRB1C and TRB2C on chr. 25 have 99% amino acid identity, with an additional amino acid in exon 1 of TRB2C. These genes belong to the same subgroup TRBC1 and were named TRB1C1-1 and TRB2C1-2 genes, respectively. By contrast, the TRB3C gene on chr. 19 shared only 70% and 69% amino acid identity with TRB1C and TRB2C, respectively. TRB3C gene belongs to the TRBC2 subgroup and was named TRB3C2-1. In *Salmo salar*, TRB1C and TRB2C belong to the TRB1C1 subgroup (TRB1C1-1 and TRB2C1-2 genes, respectively), whereas TRB3C and TRB4C belong to the TRBC2 subgroup, (TRB3C2-1 and TRB3C2-2, respectively). Significant diversity in nucleotide sequence and exon structure has been observed in TRBC genes of other teleosts (23, 24), but further studies are required to determine if they present different functional properties.

**Figure 3:**
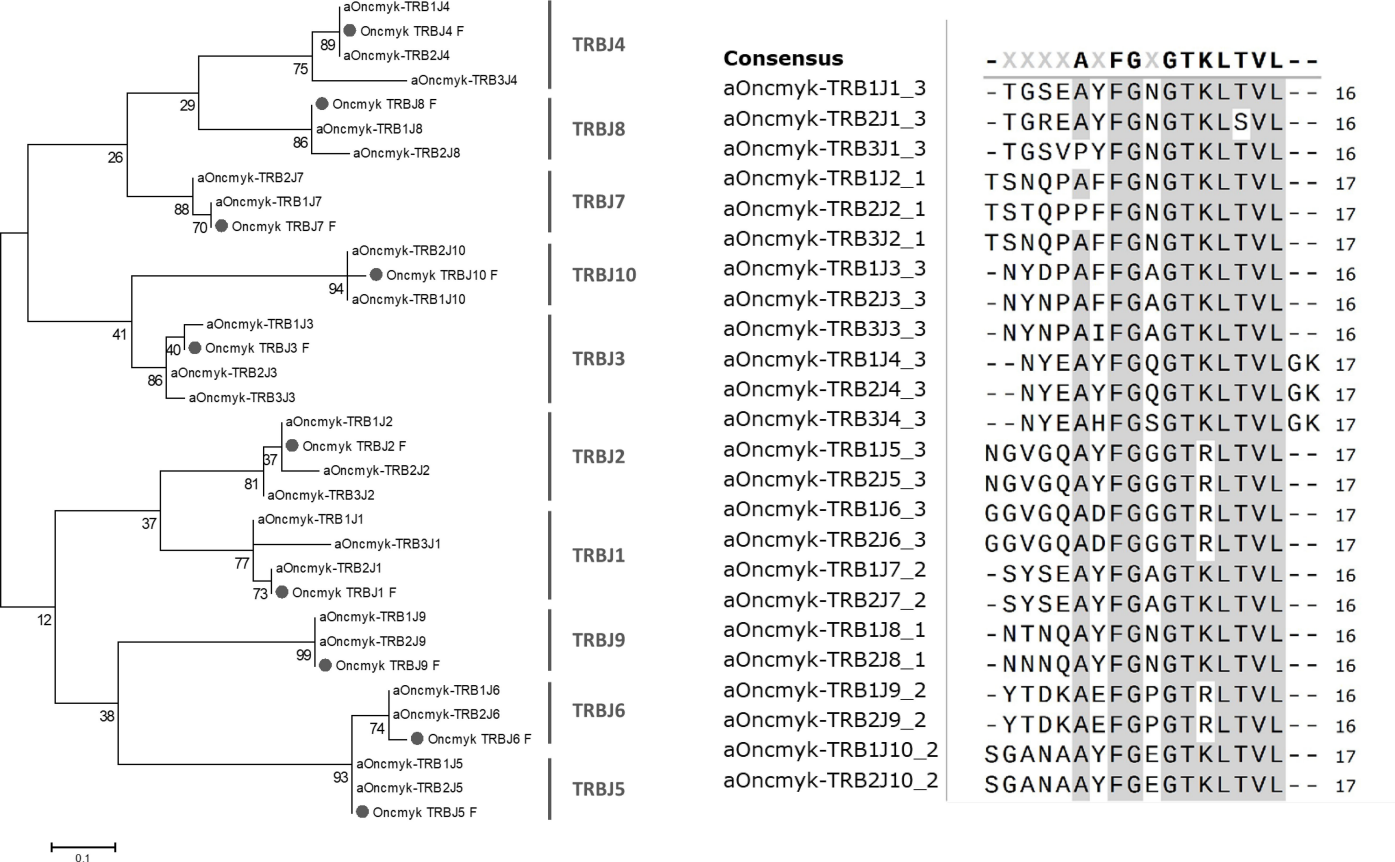
Rainbow trout TRBJ sequences. Phylogenetic tree of rainbow trout TRBJ nucleotide sequences. It includes the 24 TRBJ genes annotated in the USDA_OmykA_1.1 genome and the ten TRBJ gene sequences (labelled with a grey circle) previously identified in rainbow trout (21). The tree was inferred using the Neighbor joining method and JTT matrix based model. A bootstrap of 1000 replicates was used. Alignment of deduced amino acid sequences of rainbow trout TRBJ genes annotated in rainbow trout Arlee genome. Amino acids are in single letter code and conserved positions are grey highlighted. A consensus sequences is shown above the alignment.

Twenty TRBJ genes were found on rainbow trout chromosome 25 (**Figure 1**; **Supplementary File 2**): 10 TRBJ genes belonging to the TRB1 locus and 10 belonging to the TRB2 locus, in the opposite transcriptional orientation. Some TRBJ were represented by identical copies in inverted orientation in TRB1 and TRB2, including TRB1/2J4, -J5, -J6 and -J10. The remaining TRB1/2J genes present one or two nucleotide difference(s), that generate two (TRB1/2J1, -J2 and -J3), one (TRB1/2J8) or no (TRB1/2J9) amino acid change, as shown in **Figure 3**. In the TRB3 locus, located on chromosome 19, only 4 TRB3J genes were identified. While they do not have identical nucleotide sequences to their counterparts identified in TRB1 and TRB2 loci, two of them, TRB3J2 and TRB3J3, encode the same amino acid sequence as TRB1J2 and TRB2J3 genes, respectively (**Figure 3**). Phylogenetic analysis including the 24 TRBJ genes annotated in the USDA_OmykA_1.1 genome (**Figure 3**) reproduced all clusters defined from the ten TRBJ gene sequences previously identified in rainbow trout (21). Rainbow trout TRBJ genes show strong conservation of sequence motifs as in other vertebrates including the amino acid Phenylalanine-Glycine-X-Glycine (FGXG) J-MOTIF present in nearly all TR J-REGION [and at positions 118-121 of the V-DOMAIN (27)], as well as the conserved 6 bp splice site (GTAAGT) at the 3´end of the TRBJ genes (24, 37).

**Figure 4:**
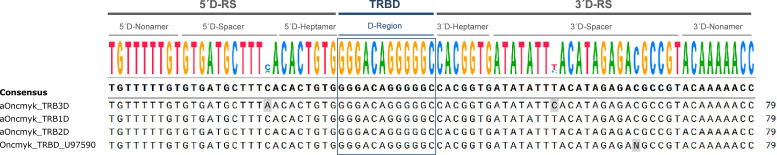
Alignment of the D-GENE-UNIT sequences of the TRBD (diversity) genes annotated in the genome of *Oncorhynchus mykiss*, USDA_OmykA_1.1 (GenBank GCA_013265735.3, isolate Arlee). The consensus sequence is provided at the top of the figure. It includes the 5’ and 3’ recombination signal (RS) sequences (5´D-RS and 3´D-RS) and the TRBD region. Oncmyk_TRBD_U97590 corresponds to the TRBD gene previously identified in rainbow trout (20).

A TRBD gene is present upstream of each TRBJ gene cluster, and it was annotated in accordance with the corresponding locus. The three TRBD gene sequences are identical, with a 12bp G-rich stretch that can be productively read in all coding frames and, depending on the frame, it encodes 1 or 3 glycines (**Figure 4**). The 5’D-RS and 3’D-RS sequences that flank the TRBD genes are well conserved between the three TRB loci, and there is even significant similarity among the 12 bp 5’D-SPACER as well as the 23 bp 3’D-SPACER.

### Rainbow trout TRBV genes

**Figure 5:**
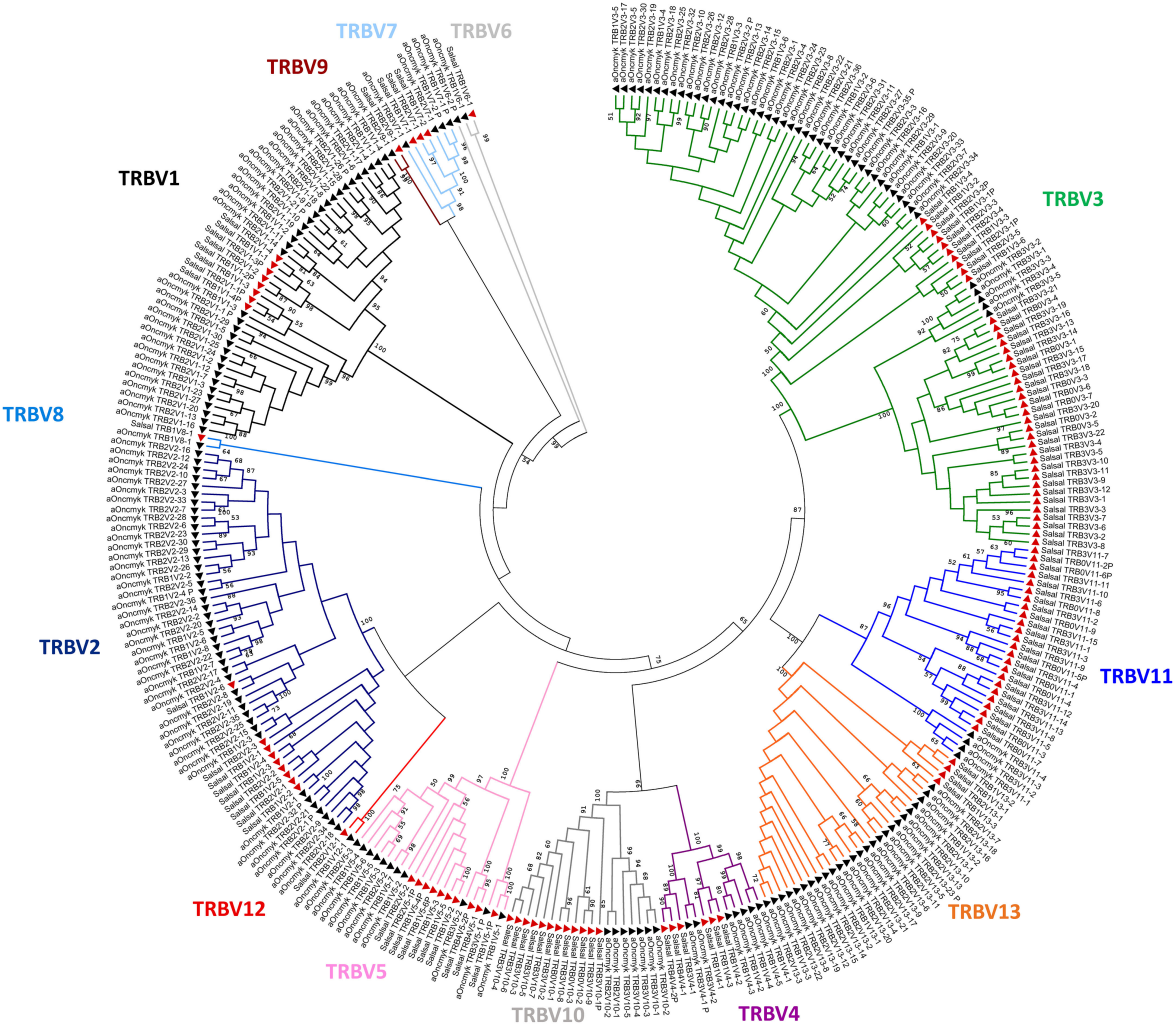
Salmonids TRBV subgroups. Phylogenetic tree of TRBV nucleotide sequences from Atlantic salmon (red triangles) and rainbow trout (black triangles). The tree was inferred using the Neighbor joining method and JTT matrix based model. A bootstrap of 1000 replicates was used. The tree with highest likelihood is shown, and is drawn to scale, with branch lengths corresponding to the number of substitutions per site. The percentage of trees in which the associated taxa cluster together is indicated when >50. The analysis has been performed with all annotated TRBV genes, functional, with open reading frame and pseudogenes (P). The subgroup branches are represented in different colors.

In the USDA_OmykA_1.1 genome, a total of 188 TRBV genes were identified, of which 172 can be considered functional or with an open reading frame (ORF) without stop codons (**Figure 1**; **Table 1**). Nineteen TRBV genes were located on chromosome 19, 17 of them in the same transcriptional orientation as the TRB3C gene, and 169 TRBV genes on chromosome 25, of which 39 and 130 were in the same transcriptional orientation as the TRB1C and TRB2C genes, respectively. As for the TRBV genes previously annotated in the Atlantic salmon genome (n=119), the 188 rainbow trout TRBV genes could be classified into the same 13 subgroups (22), as shown in a phylogenetic tree based on nucleotide sequences (**Figure 5**).

**Table 1:** TRBV genes identified in Rainbow trout _ Arlee genome.

TRBV subgroup	N° of genes Rainbow trout _Arlee (Atlantic salmon)	Rainbow trout_Arlee								
		Chromosome 25						Chromosome 19		
		TRB1			TRB2			TRB3^#^		
		F^£^	ORF	P^£^	F	ORF	P	F	ORF	P
TRBV1	34 (8)	4	0	0	24	2	4	0	0	0
TRBV2	43 (9)	7	0	1	32	1	2	0	0	0
TRBV3	47 (39)	6	0	0	34	0	2	5	0	0
TRBV4	7 (6)	5	0	0	0	0	0	1	0	1
TRBV5	11 (10)	7	0	0	2	0	0	1	0	1^#^
TRBV6	2 (1^)	1^	0	1	0	0	0	0	0	0
TRBV7	4 (3)	0	0	2*	0	1	0	1^#^	0	0
TRBV8	1 (1)	1	0	0	0	0	0	0	0	0
TRBV9	1 (1)	0	0	0	1	0	0	0	0	0
TRBV10	7 (12)	0	0	0	0	2	0	1	4	0
TRBV11	4 (24)	0	0	0	0	0	0	4	0	0
TRBV12	1 (1)	1	0	0	0	0	0	0	0	0
TRBV13	26 (4)	3	0	0	20	2	1	0	0	0
Total	188 (119)	35	0	4	113	8	9	13	4	2
		169 (148 F, 8 ORF, 13 P)						19 (13 F, 4ORF, 2P)		

* TRBV without stop codons, but pseudogenes (P) due to the lack of a V-RS. ^£^ Functional (F), if the coding region has an open reading frame, no defect in splicing site, or in RS and presents key conserved amino acids. Open Reading Frame (ORF), if the coding region has an open reading frame but there are defects in the splicing sites or RS sequences, and/or changes of conserved amino acids that lead to incorrect folding. Pseudogene (P), if the coding region has stop codon, frameshift mutations or there is no RS sequence. ^ The functional TRB1V6 gene (TRB1V6-1 in Atlantic salmon and TRB1V6-2 in rainbow trout) presents a non-conventional V-EXON with two inserts (introns) configuration. **^#^** There are 2 TRBV (**^#^**) that belong to the remnant TRB4 locus.

The number of rainbow trout TRBV genes present in each subgroup was variable, from only one gene (*i.e.* TRBV8, TRBV9 and TRBV12, all them being single functional gene of their subgroup) to 47 genes (TRBV3 subgroup with 45 functional genes) or 43 genes (TRBV2 subgroup with 39 functional genes). Though all TRBV subgroups are present in both salmonid species, the distribution of the TRBV genes per subgroup was quite different (**Table 1**). The four most represented subgroups in rainbow trout were TRBV3 (47 genes), TRBV2 (43 genes), TRBV1 (34 genes) and TRBV13 (26 genes) (**Figure 5**; **Table 1**).

**Figure 6:**
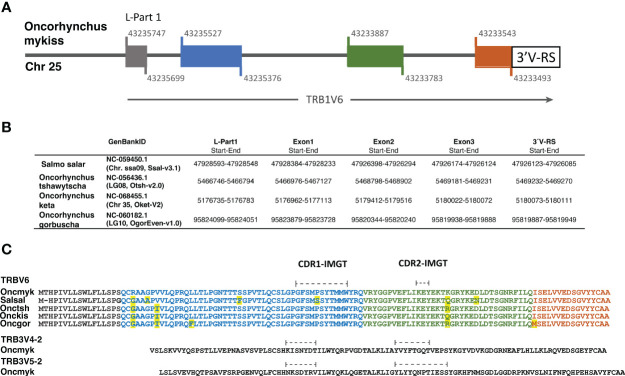
The functional TRBV6 gene comprises 4 exons and is conserved across Salmonids. **(A)** Structure of the functional TRB1V6-1 gene in the rainbow trout genome (Arlee strain; USDA OmykA_1.1, GenBank GCA_013265735.3). Colored boxes indicate exons and black line indicates introns. Nucleotide position of the exon start and exon end at the Chr. 25 is shown above and below of each box. **(B)** The localization of TRBV6 exons and 3´V-RS sequences in the genome of 4 additional salmonid species is shown in a table. In Supplementary file 3 the corresponding nucleotide sequences are available. **(C)** Alignment of TRBV6 amino acid sequences of several salmonid species: rainbow trout, Oncmyk AAN15758; Atlantic salmon, SalsalTRBV6 Genome Ssal_3.1 reference release 102. Chr09, join:47916622‐47916678; 47916813‐47916971; 47926449‐47926282; 47926180‐47926127); Chinook salmon, OnctshTRBV6: XP_042181049; Chum salmon, OnckisTRBV6: XP_052352121; Pink salmon, OncgorTRBV6: XP_046223527). The amino acid sequence encoded by each exon is shown in the corresponding color. Differences between species are yellow highlighted.

In both rainbow trout and Atlantic salmon, the method used for TRBV gene annotation first resulted in the identification of a TRBV6 subgroup comprising a single pseudogene. Further scan of rainbow trout chr. 25 and chr. 19 using tBLASTn and the published TRBV6 amino acid sequence (AY135387 (21)) as a query, led to the identification of a single genomic sequence encoding a functional TRBV6 gene, which spans from basepairs 43233493 to 43235747 in chr. 25 (**Figure 6**). Strikingly, the structure of the rainbow trout TRBV6 gene revealed peculiar features: while TRBV genes - and generally the IG and TR V genes – are composed of two exons (L-PART1 and V-EXON which encodes L-PART2 and V-REGION), the functional TRBV6 gene comprised 4 exons (**Figure 6A**), owing to the insertion of 2 introns in the V-EXON. This atypical structure was conserved across salmonids both in *Salmo* and *Oncorhynchus* species (**Figure 6B**), which excluded that the configuration seen in rainbow trout might be due to a wrong genome assembly. While only one copy of TRBV6 was found in each salmonid genome, these genes do not look pseudogenized, and sequences are highly conserved (**Figure 6C**; **Supplementary File 3**). Furthermore, the position in the locus is also preserved, as in both rainbow trout and Atlantic salmon the functional TRBV6 gene is located in between TRB1V8-1 and TRB1V7-1 genes (**Supplementary File 1**). Our previous spectratyping analyses of rainbow trout TRBV6 repertoire after viral infection further supported TRBV6 functionality, as this TRBV gene appeared to be involved in the response (21). TRBV6 was found in Refseq mRNAs encoding functional TRB rearranged sequences from brown trout (XM_029712673), chinook salmon (XM_042325115), chum salmon (XM_052496161) and pink salmon (XM_046367571). However, among RNA/EST sequences available in databases, TRBV6 was scarce suggesting that it was generally not highly expressed. In addition to our previous study in rainbow trout [(21); eg AY135387], TRBV6 was found in Coho salmon TSA [GDQG01003592]. TRBV6-like sequences were also identified in the grayling (*Thymallus thymallus*) [GFVB01062076], and *Coregonus artedi* [GIUL01081352; GIUL01081353], two other salmonids. While these data indicate that TRBV6 is expressed in several salmonid species, its usage will have to be further assessed in response to various antigens.

### Comparative analysis of TRB loci across salmonids

**Figure 7:**
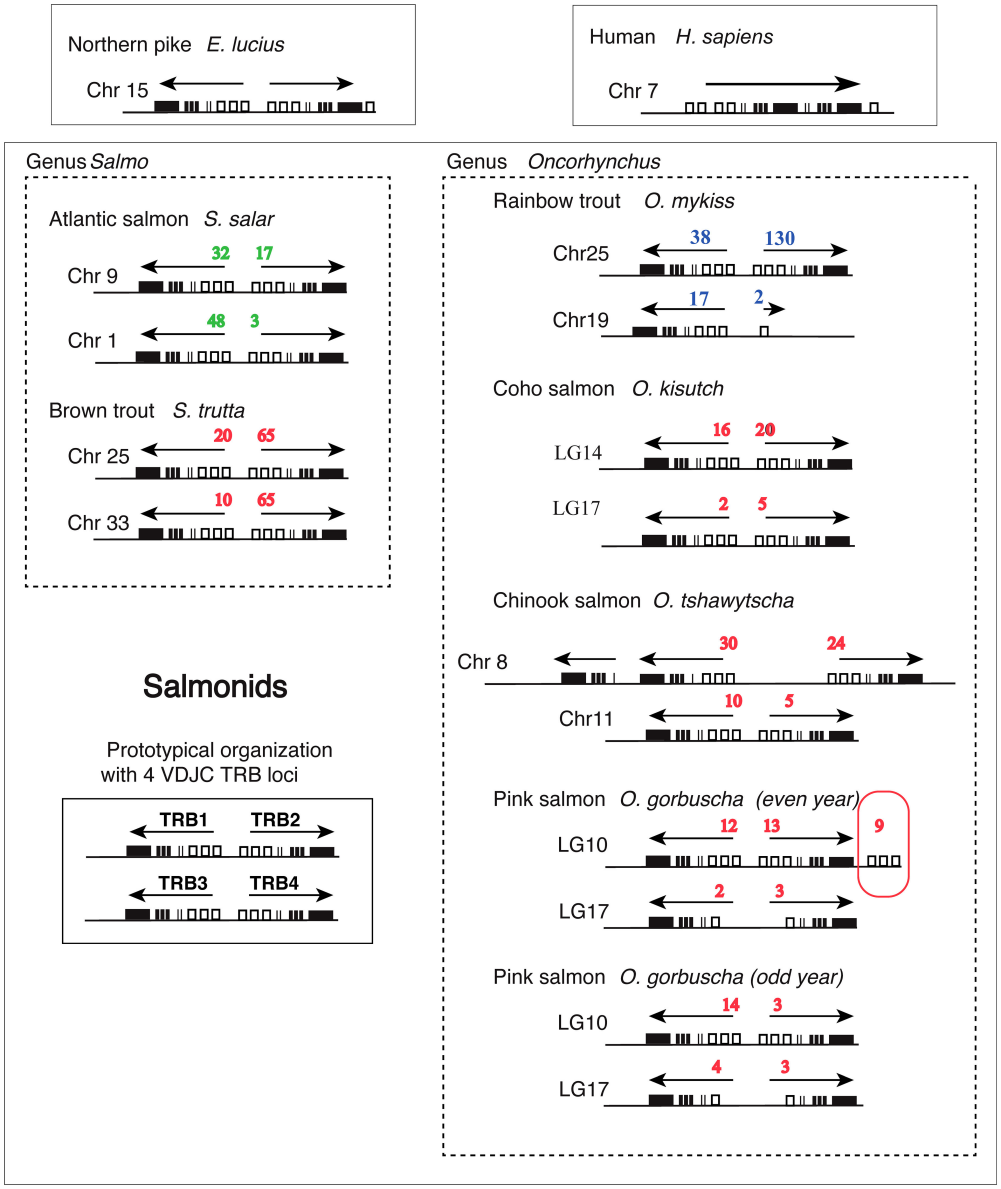
Structure of TRB loci across salmonid species. Genomic location and organization of TRB loci was determined based on last available genome assemblies at NCBI and Ensembl. Loci from rainbow trout (this work) and Atlantic salmon (22) are represented besides those from brown trout (*Salmo trutta*), Coho salmon (*Oncorhynchus kisutch*), Chinook salmon (*Oncorhynchus tshawytscha*), and Pink salmon (*Oncorhynchus gorbuscha*) (even and odd year). Northern pike (*Esox lucius*) is shown above as a representative from a sister group of Salmonids, which has not been subjected to WGD. Human (*Homo sapiens*) is shown above as a representative from mammals. White rectangles represent TRBV genes, thin lines TRBD genes, thick lines TRBJ genes and black rectangles TRBC genes. Numbers of TRBV genes (functional, ORF and pseudogenes) detected in our analysis are indicated in blue for rainbow trout, in green for Atlantic salmon and in red for the other species. Transcriptional orientation (determined by TRBC genes) is indicated above each locus.

The presence of different numbers of TRB loci in Atlantic salmon and rainbow trout led us to further investigate the genomic organization of TRB loci and genes in different salmonid species. TRBV, -D, -J and -C sequences from rainbow trout were used in blast analyses to identify their counterparts in brown trout (*Salmo trutta*), chinook salmon (*Oncorhynchus tshawytscha*), coho salmon (*Oncorhynchus kisutsh*), and pink salmon even and odd year (*Oncorhynchus gorbutscha*). We also included Northern pike (*Esox lucius*) that split from the salmonid lineage prior to the ssWGD. The comparison of TRB locus structure across pike, rainbow trout, Atlantic salmon and these species (**Figure 7**; **Supplementary File 4**) reveals a clear pattern. The basic structure of salmonid TRB is a set of two V-D-J-C loci duplicated head-to-head, in opposite orientation with TRBV genes in the middle (**Figure 7**), which is already present in northern pike on a single chromosome (chr 15). Two sets of duplicated loci are typically present in salmonid genomes, located on different chromosomes. Thus, they likely resulted from the ssWGD, with variations due to later events. Despite the absence of a TRB4 D-J-C cluster in rainbow trout, two TRBV genes in inverted orientation were assigned to TRB4, while in chinook salmon a local duplication of a D-J-C segment led to a [C-J-D]-[C-J-D-V]-[V-D-J-C] structure (**Figure 7**). Two sets of duplicated loci were found in all other analyzed species of salmonids. Within a given [V-D-J-C] locus, almost all genes were in the same orientation, suggesting they constitute translocon recombination units. The only exception was a set of 9 V genes on LG10 of the even year pink salmon genome, which were located downstream of the TRB2C gene, in forward orientation (circled in red in **Figure 7**). However, this was most likely due to an assembly issue, as this region is made of many small contigs.

**Figure 8:**
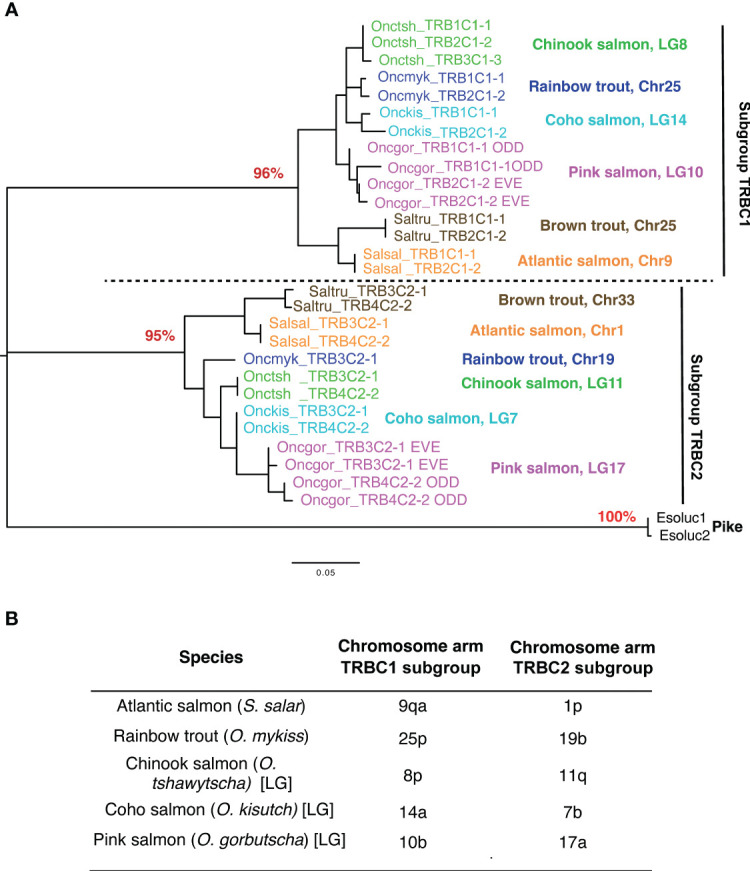
TRBC subgroups. **(A)** Phylogenetic analysis of TRBC genes reveals two distinct types located on different chromosomes in each salmonid species. These types have been shown to correspond to two TRBC subgroups, C1 and C2. The TRBC1 subgroup includes TRB1C1-1 and TRB2C1-2 and the TRBC2 subgroup includes TRB3C2-1 and TRBC4C2-2. Phylogenetic reconstruction was inferred by using the Maximum Likelihood method and JTT matrix-based model. The tree with the highest log likelihood (-1537.90) is shown. The percentage of trees in which the associated taxa clustered together is shown next to the key nodes corresponding to types A and B as a percentage (in red). Evolutionary analyses were conducted in MEGA X. The tree was rooted by TRBC sequences from the Northern pike. **(B)** TRB loci are found on distinct chromosome arms generated by the ssWGD. The location of TRB loci is consistent with chromosomal orthology relationships between species.

Strikingly, TRBC genes were highly similar between duplicated loci located on the same chromosome, while sequences from different chromosomes grouped with high-bootstrap values into two different TRBC subgroups in phylogenetic analyses (**Figure 8A**; **Supplementary File 5**). The TRB1C, TRB2C, TRB3C and TRB4C gene names have been upgraded for repertoire analyses with the subgroup number, followed by a dash and a number in the subgroup for the repertoire analyses: TRB1C1-1, TRB2C1-2 (chr. 25 Oncmyk, chr. 9 Salsal), TRB3C2-1 (chr. 19 Oncmyk, chr. 1 Salsal) and TRB4C2-2 (chr. 1 Salsal), as predicted by chromosomal orthology (38–40) (**Figure 8B**).

V-D-J-C loci on rainbow trout chromosome 25 comprised more TRBV genes than the one on chromosome 19. This pattern was found in all the other *Oncorhynchus* species we examined, with varying number of TRBV genes (**Figure 7**).

### Expression of different TRBC subgroups

It has been previously reported for Atlantic salmon that TRBC1 [“type A” in (22)], was significantly more expressed than TRBC2 [“type B” in (22)]. Reads from available datasets of spleen transcriptomes were mapped to TRBC1 or TRBC2, of respectively rainbow trout, coho salmon, chinook salmon, and pink salmon. As in Atlantic salmon, TRBC1 sequences were more expressed than TRBC2 in all these species, with TRBC1/TRBC2 ratios varying from 2.3 to 7 (**Table 2**; **Supplementary File 4**). The same trend was found from a multi-tissue dataset from brown trout. Hence, the dominant expression of TRBC1 subgroup over TRBC2 subgroup appears to be a conserved feature across salmonids from *Salmo* and *Oncorhynchus* genera.

**Table 2:** Expression of TRBC1 and TRBC2 subgroups across salmonid species.

Species	Dataset ID	Tissue	Input reads	RPKM TRBC1	RPKM TRBC2	ratioRPKM TRBC1/2
*O. tshawytscha* TRBC1: Chr.8 TRBC2: Chr.11	SRR6273681	spleen	110639563	45,7	6,7	6,8
	SRR3986817	spleen	14921851	72,5	10,0	7,3
	SRR3986815	spleen	16726316	68,0	11,3	6,0
*O. kisutch* TRBC1: Chr14 TRBC2: Chr 7	SRR5333378	spleen	70389516	171,8	70,8	2,4
	SRR5333374	spleen	76816418	208,6	62,4	3,3
	SRR5333360	spleen	66842954	150,4	48,2	2,6
*O. mykiss* TRBC1: Chr 25 TRBC2: Chr 19	SRR11972660	spleen	1494649	47,9	20,5	2,3
	SRR11972662	spleen	687545	79,9	24,4	3,3
	SRR11972726	spleen	1036767	115,4	41,0	2,8
*O. gorbutscha* TRBC1: Chr 10 TRBC2: Chr 17	SRR9841114	spleen	83100156	150,4	48,2	3,1
*S. trutta* TRBC1: Chr25 TRBC2: Chr33	SRR6666113	Multi tissues*	46960387	132,4	14,3	9,3

* Fresh brain, heart, gill, liver, intestines, muscle, gonads (ovary and testis), kidney, spleen and eye.

## Discussion

A full annotation of the TRB locus has been reported in only a few fish species, including zebrafish, channel catfish, and recently the Atlantic salmon (22–24). In the latter species, TRB genes were reported to be organized in two sets of inverted duplicated TRB V-D-J-C loci, located on two different chromosomes. We present here a full description of the germline configuration of TRB genes in the genome of another salmonid, the rainbow trout. High-quality genome assemblies for several *Oncorhynchus* and *Salmo* species provide the opportunity to compare the structure and composition of their TRB loci. In addition to the teleost-specific WGD, a second WGD occurred in early Salmonids and a high proportion (about 50%) of the resulting paralog pairs has been retained, generating a large molecular diversity (11). We discuss here how TRB loci and gene diversity evolved in Salmonids. In the present work, we updated and completed the genomic annotation of the rainbow trout TRB loci based on the sequence of the whole genome assembly USDA_OmykA_1.1 (GCA_013265735.3), using as a reference the classification of Atlantic salmon TRB genes proposed in (22). The rainbow trout genome analysis revealed the presence of three TRB V-D-J-C loci located on chr. 19 and chr. 25. In contrast to what was described in Atlantic salmon with two duplicated TRB V-D-J-C loci, there was only one complete set of duplicated TRB V-D-J-C (comprising the TRB1 and TRB2 loci) in rainbow trout, located on chromosome 25; and only one full TRB V-D-J-C locus on rainbow trout chr. 19, however the two TRBV genes in inverted orientation on chr.19 most probably belong to a TRB4 locus lost during evolution. The number of annotated TRBV genes also differed between these two salmonid species, being 188 in rainbow trout and 119 in Atlantic salmon, but they cluster in the same thirteen TRBV subgroups. The major germline rainbow trout TRB repertoire was attributable to the expansion of four TRBV subgroups (TRBV1, -2, -3 and -13), whose functional genes represent 71% of the total TRBV repertoire. The TRBV3 subgroup was also expanded in Atlantic salmon which, along with TRBV11, -10 and -5, corresponded to the most expanded subgroups in this salmonid species (see **Table 1**). An analogous situation was previously described in rainbow trout and Atlantic salmon IGH and TRA/D loci, that present different numbers of variable genes in both species, which cluster in similar numbers of subgroups but with different distributions (14, 15). Therefore, as described in other vertebrates, expansions of individual gene subgroups, rather than the emergence of novel sequences, seems to be the major mode of evolution of salmonid IG and TR variable genes (9, 14, 41, 42).

Strikingly, the numbers of TRBV pseudogenes was lower in rainbow trout compared to Atlantic salmon: 8% (15 out of 188 TRBV genes) versus 15% (18 out of 119 TRBV genes) in Atlantic salmon (22). This trend was consistent with the percentage of pseudogenes found in the IGH and TRA/D loci (14, 15) of these species: 28,5% of pseudogenes among rainbow trout IGHV versus 73% in Atlantic salmon (14), and 34% of pseudogenes among rainbow trout TRA/DV versus 51% in Atlantic salmon (15). It is also worth noting that the proportion of pseudogenes among salmonid TRBV genes is particularly low, while the opposite trend is observed in human (29%, *i.e.* 19 among 65 TRBV) for (18%, i.e. 10 among 54 TRA/DV). These differences in pseudogene frequencies between species and between IG and TR antigen receptor genes might result from differential rates of mutation and/or pseudogene elimination. It is not clear whether these differences are due to contrasting genomic dynamics, or to selection pressures related to the T cell response against specific pathogens.

Among salmonid TRBV genes, TRBV6 stands out with a particular genomic structure and atypical characteristics of its variable domain. In the five salmonid species tested here, this TRBV gene contained 4 exons: one for the leader peptide, and 3 exons coding for the variable region. While the expression of diverse rearrangements involving TRBV6 was previously reported in rainbow trout, both in controls and virus infected fish (21), its remarkable structural features – combining a long CDR1-IMGT (9 AA) and a short and highly hydrophilic CDR2-IMGT (3AA) – might point to a particular mode of peptide recognition. Taken together with the peculiar CDRs of salmonid TRA/DV, this structural diversity of TRBV may question the typical 3D-configuration of the TR/peptide/MHC tricomplex. Future research should investigate the contribution of TRBV6 in the TRB repertoire, as well as its combination with TRA/DV chains showing unusual structural features such as TRAV22, and TRAV3 that also lacks CDR2 (15).

Rainbow trout and Atlantic salmon TRBC genes from duplicated loci present on a given chromosome encode near identical products, reflecting a minimal divergence following inversion/duplication. Comparison of TRBC gene sequences from different chromosomes revealed two different TRBC subgroups based on IMGT rules, which we named TRBC1 and 2. This was a conserved feature across analyzed salmonids from *Salmo* and *Oncorhynchus* genera. Both subgroups were encoded by four exons and share around 70% of amino acid sequence identity. Unlike in mammalian species, where all described TRBC genes are very similar, since they differ by only a few amino acids in the coding region (9), in teleosts and other non-mammal vertebrates a number of divergent subgroups have been described. For example, in channel catfish there are two tandem TRBC genes that share only 36% identity at the amino acid level (23, 26). Divergent TRBC genes were also identified in the bicolor damselfish (*Stegastes partitus*) (43, 44). In amphibians, the Mexican axolotl appears to have multiple divergent TRBC genes as assessed by cDNA analysis (45). In contrast to genes encoding the TRB variable domain that bind to a highly diverse array of peptide-MHC ligands, TRBC gene products interact with the extracellular part of the TRA chain (46) and with the non-polymorphic components of the CD3 coreceptor. These interactions condition the association and surface expression of the functional T cell receptor (i.e., the TR-CD3 complex), and play an important role in signal transduction (47). The functional capacity and specificity of each salmonid TRBC subgroup in combination with available CD3 chains, remains an open question. Nevertheless, the concerted evolution of the TRBC genes that seems to be a requirement in mammalian species is not so evident in other vertebrates. Furthermore, different transcription patterns for salmonid TRBC subgroups were observed, where TRBC1 expression dominated over TRBC2 in all studied species. V-D-J-C loci expressing TRBC1 comprise more TRBV genes, which reflect a higher potential diversity of antigen recognition sites. However, further studies are required to clarify if the different salmonid TRBC subgroups or genes may confer any particular biological properties or functional significance.

Another important question is the impact of multiple TRB loci on allelic exclusion. The differentiation of human and mice T and B lymphocytes, is a highly regulated process, wherein the generation of productive specific antigen receptors follows the so called “ordered” model (48). The expression of an unique clonal receptor by each lymphocyte is critical to warrant a unique specificity of the antigen receptor expressed by a given lymphocyte, one of the bases of the clonal selection theory (49, 50). The differentiation of a pro T lymphocyte into pre T requires a productive rearrangement at the TRB locus, leading to the expression of the pre-T-cell receptor (pre-TR), consisting of the TRB product of this rearrangement linked to the invariant pre-T alpha (pTα) chain encoded by an unrearranged gene (50, 51) located outside of TR loci. The signaling through the pre-TR ensures allelic exclusion, stopping rearrangement at the TRB locus and starting the process at the TRA locus (52). Interestingly, a pTα gene has been identified in mammals and in the chicken, but not in amphibians or fish (53). Although a recent study with CD79- green fluorescent protein (GFP) transgenic zebrafish lines suggests that a surrogate L chain might not be required for B cell development in this species (54), very little is known about T cell maturation and its kinetics in teleost. The mechanisms ensuring the clonal expression of the T cell receptor by salmonid αβ T cells, with four loci present in each haplotype, remain unknown. In mammalian B cell precursors, an IgH “holo-complex” consisting of nuclear factors that bind IGHV regulatory sequences, induce chromatin remodeling and open the region to RAG mediated recombination (55). The epigenetic control of allelic and locus exclusion in Salmonids may follow probabilistic models as those proposed by Hsu and co-workers to explain allelic exclusion in the context of multiple IgH miniloci in sharks (56, 57), and by Schlissel et al. for mouse Ig*κ* loci (58): the accessibility of TRB genes might be dependent on the formation of an “holocomplex” at very low frequency due to limiting amounts of nuclear factors. Future characterization of TRB loci rearrangements and TRB mRNA expression at single cell level will shed light on these mechanisms.

## Conclusions

In conclusion, we have provided comprehensive annotation and nomenclature for the rainbow trout multiple TRB loci. This work paves the way for TRB repertoire analysis in this species and provides insights about TRB evolution in Salmonids.

## Data availability statement

The datasets presented in this study can be found in online repositories. The names of the repository/repositories and accession number(s) can be found in the article/**Supplementary Material**.

## Author contributions

PB and SuM conceived the project, SN, LJ, StM, M-PL, UG, PB and SuM performed data analysis. PB, M-PL, UG and SuM wrote the manuscript. All authors contributed to manuscript revision, read and approved the submitted version.
