# Supplementary material for: Evolution of T cell receptor beta loci in salmonids
Source: Front Immunol. 2023 Aug 15;14:1238321. doi: 10.3389/fimmu.2023.1238321 (PMC10464911; doi:10.3389/fimmu.2023.1238321)
Supplement: Supplementary file 1 [file DataSheet_1.pdf]

Supplementary File 1

IMGT-NC approved nomenclature of Atlantic salmon and rainbow trout TRB genes.

The coordinates consider the recombination signal (RS) sequence and coding regions of TRBV, TRBJ, and TRBD genes, and refer to the genomic region that contains the 4 exons of the TRBC genes. The correspondence between IMGT nomenclature and the gene name, which has been used in the previous work about Atlantic salmon TRB genes (22) is shown. Data are from (33) for Atlantic salmon (*Salmo salar*) and from (34) for rainbow trout (*Oncorhynchus mykiss*) Arlee.

An update has been made to the localization of Oncmyk TRB1V6-1, the revised coordinates of which are 43235747.. 43233493, placing this gene between TRB1V8-1 and TRB1V7-1 genes, similarly to the localization of TRB1V6-1 in the Salsal genome assembly (IMGT-NC approval, 14/05/2023).

The previously rainbow trout TRB3V7-1 and TRB3V5-1 genes, have been shown to be a reminiscence of TRB4 locus, and have been assigned to the IMGT gene names TRB4V7-1 and TRB4V5-1.

Salmo salar TRB loci sequences (annotation of genome assembly: GCA_905237065.2)

3 sequences (163 genes) corresponding to _Salmo salar_ (Atlantic salmon) T cell receptor beta (TRB) loci on chr. 9 and chr. 1:

NC_059450.1 (chr. 9) containing for the **TRB1** locus a total of 44 genes: 32 TRB1V genes (23 F, 2 O and 7 P), 1 TRB1D, 10 TRB1J and 1 TRB1C genes, and for the **TRB2** locus a total of 29 genes: 17 TRB2V genes (12 F, 5 P), 1 TRB2D, 10 TRB2J and 1 TRB2C genes.

NC_059442.1 (chr. 1) containing for the **TRB3** locus a total of 59 genes: 48 TRB3V genes (45 F, 1 O, 2 P), 1 TRB3D, 9 TRB3J and 1 TRB3C, and for the **TRB4** locus a total of 12 genes: 3 TRB4V genes (2 F, 1 P), 1 TRB4D, 7 TRB4J and 1 TRB4C.

NW_025550634.1 scaffold (TRB0) containing 19 TRB0V (16 F, 3P).

The 163 genes are **NEW** and assigned to **allele *01**. Functionality may be modified following detailed biocuration.

***Salmo salar* (Atlantic salmon) T cell receptor beta (TRB) genes on chr. 9**
(GCA_905237065. 2: TRB1 (TRB09R) and TRB2 (TRB09), NC_059450.1.

TRB1 (REV)				
IMGT gene names	Gene names	Gene orientation in locus	Genomic coordinates on *Salmo salar* chr. 9	Functionality
TRB1V13-1	TRB09RV13-1	direct	NC_059450.1:48317944-48318299	O
TRB1V3-1	TRB09RV3-1_P	direct	NC_059450.1:48315955-48316433	P
TRB1V2-1	TRB09RV2-1_F	direct	NC_059450.1:48314081-48314588	F*
TRB1V1-1	TRB09RV1-1	direct	NC_059450.1:48296403-48296987	O
TRB1V3-2	TRB09RV3-2_F	direct	NC_059450.1:48294060-48294560	F*
TRB1V13-2	TRB09RV13-2	direct	NC_059450.1:48284689-48285590	F
TRB1V3-3	TRB09RV3-3_F	direct	NC_059450.1:48283347-48283847	F*
TRB1V2-2	TRB09RV2-2	direct	NC_059450.1:48278884-48279401	F
TRB1V1-2	TRB09RV1-2_P	direct	NC_059450.1:48266327-48266910	P
TRB1V3-4	TRB09RV3-4_F	direct	NC_059450.1:48263635-48264135	F
TRB1V2-3	TRB09RV2-3_F	direct	NC_059450.1:48257219-48257742	F*
TRB1V5-1	TRB09RV5-1_P	direct	NC_059450.1:48253944-48254285	P
TRB1V5-2	TRB09RV5-2_F	direct	NC_059450.1:48253135-48253657	F*
TRB1V5-3	TRB09RV5-3_F	direct	NC_059450.1:48250909-48251419	F*
TRB1V4-1	TRB09RV4-1_F	direct	NC_059450.1:48250113-48250682	F*
TRB1V2-4	TRB09RV2-4	direct	NC_059450.1:47981989-47982533	F
TRB1V1-3	TRB09RV1-3_F	direct	NC_059450.1:47976007-47976588	F*
TRB1V3-5	TRB09V3-5_F	**opposite**	NC_059450.1:47972414-47972916	F*
TRB1V2-5	TRB09RV2-5_F	direct	NC_059450.1:47968900-47969418	F*
TRB1V1-4	TRB09RV1-4_P	direct	NC_059450.1:47956868-47957200	P
TRB1V13-3	TRB09RV13-3_F	direct	NC_059450.1:47955887-47956502	F*
TRB1V3-6	TRB09RV3-6_F	direct	NC_059450.1:47953924-47954424	F*
TRB1V2-6	TRB09RV2-6	direct	NC_059450.1:47951133-47951659	F
TRB1V5-4	TRB09RV5-4_P	direct	NC_059450.1:47947572-47948085	P
TRB1V4-2	TRB09RV4-2_F	direct	NC_059450.1:47946774-47947356	F*
TRB1V5-5	TRB09RV5-5_F	direct	NC_059450.1:47945965-47946487	F*
TRB1V5-6	TRB09RV5-6_P	direct	NC_059450.1:47943754-47944264	P
TRB1V4-3	TRB09RV4-3_F	direct	NC_059450.1:47942950-47943532	F*
TRB1V8-1	TRB09RV8-1_F	direct	NC_059450.1:47929696-47930249	F*
TRB1V6-1	TRB09RV6-1_F	direct	NC_059450.1:47926085-47928593	F
TRB1V7-1	TRB09RV7-1	direct	NC_059450.1:47924721-47925199	F
TRB1V7-2	TRB09RV7-2_F	direct	NC_059450.1:47918944-47919547	F*
TRB1D	TRB09RD	direct	NC_059450.1:47738982-47738902	
TRB1J1	TRB09RJ1	direct	NC_059450.1:47738463-47738383	
TRB1J2	TRB09RJ2	direct	NC_059450.1:47738284-47738203	
TRB1J3	TRB09RJ3	direct	NC_059450.1:47738067-47737987	
TRB1J4	TRB09RJ4	direct	NC_059450.1:47737855-47737778	
TRB1J5	TRB09RJ5	direct	NC_059450.1:47737349-47737266	

TRB1J6	TRB09RJ6	direct	NC_059450.1:47737117-47737034	
TRB1J7	TRB09RJ7	direct	NC_059450.1:47736701-47736622	
TRB1J8	TRB09RJ8	direct	NC_059450.1:47735123-47735045	
TRB1J9	TRB09RJ9	direct	NC_059450.1:47734779-47734700	
TRB1J10	TRB09RJ10	direct	NC_059450.1:47734436-47734354	
TRB1C	TRB09RC	direct	NC_059450.1:47729847-47732471	

F* defines sequence with expressed support.

Locus TRB2 (FWD)				
IMGT gene names	Gene names	Gene orientation in locus	Genomic coordinates on *Salmo salar* chr. 9	Functionality
TRB2V1-1	TRB09V1-1_P	direct	NC_059450.1:48398141-48398721	P
TRB2V3-1	TRB09V3-1_FP	direct	NC_059450.1:48400807-48401306	P
TRB2V2-1	TRB09V2-1	direct	NC_059450.1:48406946-48407473	F
TRB2V1-2	TRB09V1-2_F	direct	NC_059450.1:48411891-48412475	F*
TRB2V13-1	TRB09V13-1	direct	NC_059450.1:48412907-48413671	F
TRB2V3-2	TRB09V3-2_P	direct	NC_059450.1:48414812-48415307	P
TRB2V1-3	TRB09V1-3_P	direct	NC_059450.1:48427834-48428370	P
TRB2V3-3	TRB09V3-3_F	direct	NC_059450.1:48429905-48430405	F*
TRB2V2-2	TRB09V2-2_F	direct	NC_059450.1:48433151-48433675	F*
TRB2V1-4	TRB09V1-4_F	direct	NC_059450.1:48435685-48436269	F*
TRB2V3-4	TRB09V3-4_F	direct	NC_059450.1:48437952-48438452	F*
TRB2V2-3	TRB09V2-3_F	direct	NC_059450.1:48442933-48443510	F*
TRB2V5-1	TRB09V5-1_P	direct	NC_059450.1:48448032-48448547	P
TRB2V5-2	TRB09V5-2_F	direct	NC_059450.1:48452611-48453121	F*
TRB2V12-1	TRB09V12-1	direct	NC_059450.1:48460217-48460835	F
TRB2V9-1	TRB09V9-1_F	direct	NC_059450.1:48463790-48464857	F*
TRB2V7-1	TRB09V7-1_F	direct	NC_059450.1:48472720-48473323	F*
TRB2D	TRB09D	direct	NC_059450.1:48488404-48488483	
TRB2J1	TRB09J1	direct	NC_059450.1:48488963-48489043	
TRB2J2	TRB09J2	direct	NC_059450.1:48489142-48489223	
TRB2J3	TRB09J3	direct	NC_059450.1:48489358-48489438	
TRB2J4	TRB09J4	direct	NC_059450.1:48489570-48489647	
TRB2J5	TRB09J5	direct	NC_059450.1:48490076-48490159	
TRB2J6	TRB09J6	direct	NC_059450.1:48490308-48490391	
TRB2J7	TRB09J7	direct	NC_059450.1:48490725-48490804	
TRB2J8	TRB09J8	direct	NC_059450.1:48492236-48492314	
TRB2J9	TRB09J9	direct	NC_059450.1:48492579-48492658	
TRB2J10	TRB09J10	direct	NC_059450.1:48492935-48493017	
TRB2C	TRB09C	direct	NC_059450.1:48494760-48497562	

***Salmo salar* (Atlantic salmon) T cell receptor beta (TRB) genes on chr. 1**
(GCA_905237065.2, TRB3 (TRB01R) and TRB4 (TRB01), NC_059442.1;
and TRB0 (Scf.R). NW_025550634.1 although unknown placement in region.

Locus TRB3 (REV)				
IMGT gene names	Gene names	Gene orientation in locus	Genomic coordinates on *Salmo salar* chr. 1	Functionality
TRB3V11-1	TRB01RV11-1_F	direct	NC_059442.1:4383356-4383875	F*
TRB3V3-1	TRB01RV3-1	direct	NC_059442.1:4380191-4380694	F
TRB3V10-1	TRB01RV10-1_P	direct	NC_059442.1:4370777-4371033	P
TRB3V3-2	TRB01RV3-2_F	direct	NC_059442.1:4369561-4370064	F*
TRB3V10-2	TRB01RV10-2_F	direct	NC_059442.1:4362703-4363203	F*
TRB3V3-3	TRB01RV3-3_F	direct	NC_059442.1:4361487-4361990	F*
TRB3V10-3	TRB01RV10-3	direct	NC_059442.1:4353950-4354450	F*
TRB3V3-4	TRB01RV3-4	direct	NC_059442.1:4352734-4353237	F
TRB3V10-4	TRB01RV10-4	direct	NC_059442.1:4342385-4342885	F
TRB3V3-5	TRB01RV3-5	direct	NC_059442.1:4341169-4341672	F
TRB3V10-5	TRB01RV10-5	direct	NC_059442.1:4332138-4332638	F
TRB3V3-6	TRB01RV3-6	direct	NC_059442.1:4330922-4331425	F
TRB3V10-6	TRB01RV10-6	direct	NC_059442.1:4323475-4323975	F
TRB3V3-7	TRB01RV3-7	direct	NC_059442.1:4322259-4322762	F
TRB3V10-7	TRB01RV10-7	direct	NC_059442.1:4312932-4313432	F
TRB3V3-8	TRB01RV3-8_F	direct	NC_059442.1:4310278-4310781	F*
TRB3V11-2	TRB01RV11-2	direct	NC_059442.1:4300808-4301433	F
TRB3V3-9	TRB01RV3-9	direct	NC_059442.1:4296117-4296620	F
TRB3V3-10	TRB01RV3-10	direct	NC_059442.1:4293276-4293779	F
TRB3V3-11	TRB01RV3-11	direct	NC_059442.1:4290434-4290937	F
TRB3V11-3	TRB01RV11-3_F	direct	NC_059442.1:4288153-4288716	F*
TRB3V5-1	TRB01RV5-1_P	direct	NC_059442.1:4280602-4280982	P
TRB3V3-12	TRB01RV3-12	**opposite**	NC_059442.1:4266119-4266628	O
TRB3V10-8	TRB01RV10-8	direct	NC_059442.1:3892782-3893282	F
TRB3V3-13	TRB01RV3-13	direct	NC_059442.1:3891736-3892239	F
TRB3V10-9	TRB01RV10-9	direct	NC_059442.1:3889922-3890422	F
TRB3V3-14	TRB01RV3-14	direct	NC_059442.1:3888876-3889379	F
TRB3V11-4	TRB01RV11-4	direct	NC_059442.1:3887111-3887706	F
TRB3V3-15	TRB01RV3-15	direct	NC_059442.1:3882088-3882592	F
TRB3V11-5	TRB01RV11-5	direct	NC_059442.1:3875077-3875665	F
TRB3V11-6	TRB01RV11-6_F	direct	NC_059442.1:3870456-3870997	F*
TRB3V3-16	TRB01RV3-16	direct	NC_059442.1:3865434-3865938	F
TRB3V11-7	TRB01RV11-7	direct	NC_059442.1:3855984-3856523	F
TRB3V3-17	TRB01RV3-17	direct	NC_059442.1:3850984-3851488	F
TRB3V11-8	TRB01RV11-8	direct	NC_059442.1:3843979-3844565	F
TRB3V11-9	TRB01RV11-9	direct	NC_059442.1:3839175-3839770	F
TRB3V11-10	TRB01RV11-10_F	direct	NC_059442.1:3834337-3834878	F*

TRB3V11-11	TRB01RV11-11	direct	NC_059442.1:3827468-3828008	F
TRB3V3-18	TRB01RV3-18	direct	NC_059442.1:3822450-3822954	F
TRB3V11-12	TRB01RV11-12	direct	NC_059442.1:3815438-3816026	F
TRB3V3-19	TRB01RV3-19	direct	NC_059442.1:3811077-3811581	F
TRB3V11-13	TRB01RV11-13	direct	NC_059442.1:3804025-3804613	F
TRB3V3-20	TRB01RV3-20_F	direct	NC_059442.1:3799669-3800173	F*
TRB3V11-14	TRB01RV11-14	direct	NC_059442.1:3792273-3792869	F
TRB3V3-21	TRB01RV3-21	direct	NC_059442.1:3787918-3788415	F
TRB3V11-15	TRB01RV11-15_F	direct	NC_059442.1:3780795-3781324	F*
TRB3V3-22	TRB01RV3-22_F	direct	NC_059442.1:3775809-3776312	F*
TRB3V4-1	TRB01RV4-1	direct	NC_059442.1:3751052-3751641	F
TRB3D	TRB01RD	direct	NC_059442.1:3725066-3725145	
TRB3J1	TRB01RJ1	direct	NC_059442.1:3716388-3716308	
TRB3J2	TRB01RJ2	direct	NC_059442.1:3716206-3716125	
TRB3J3	TRB01RJ3	direct	NC_059442.1:3715980-3715900	
TRB3J4	TRB01RJ4	direct	NC_059442.1:3715768-3715691	
TRB3J5	TRB01RJ5	direct	NC_059442.1:3715465-3715382	
TRB3J6	TRB01RJ6	direct	NC_059442.1:3714953-3714874	
TRB3J7	TRB01RJ7	direct	NC_059442.1:3714143-3714065	
TRB3J8	TRB01RJ8	direct	NC_059442.1:3713788-3713709	
TRB3J9	TRB01RJ9	direct	NC_059442.1:3713361-3713279	
TRB3C	TRB01RC	direct	NC_059442.1:3711729-3712624	

Locus TRB4 (FWD)				
IMGT gene names	Gene names	Gene orientation in locus	Genomic coordinates on *Salmo salar* chr. 1	Functionality
TRB4V4-1	TRB01V4-1	direct	NC_059442.1:4392054-4392949	F*
TRB4V5-1	TRB01V5-1	direct	NC_059442.1:4393250-4393812	F
TRB4V4-2	TRB01V4-2_P	direct	NC_059442.1:4405432-4406100	P
TRB4D	TRB01D	direct	NC_059442.1:4424590-4424669	
TRB4J1	TRB01J1	direct	NC_059442.1:4427676-4427756	
TRB4J2	TRB01J2	direct	NC_059442.1:4427858-4427939	
TRB4J3	TRB01J3	direct	NC_059442.1:4428084-4428164	
TRB4J4	TRB01J4	direct	NC_059442.1:4428296-4428373	
TRB4J5	TRB01J5	direct	NC_059442.1:4428599-4428682	
TRB4J6	TBR01J6	direct	NC_059442.1:4429083-4429162	
TRB4J7	TRB01J7	direct	NC_059442.1:4429841-4429923	
TRB4C	TRB01C	direct	NC_059442.1:4431094-4431989	

Locus TRB0 (REV)				
IMGT gene names	Gene names	Gene orientation in locus	Genomic coordinates of *Salmo salar* TRBV genes on scaffold NW_025550634.1	Functionality
TRB0V10-1	Scf.RV10-1	direct	NW_025550634.1:90517-91017	F
TRB0V3-1	Scf.RV3-1	direct	NW_025550634.1:89471-89974	F
TRB0V10-2	Scf.RV10-2	direct	NW_025550634.1:87657-88157	F
TRB0V3-2	Scf.RV3-2	direct	NW_025550634.1:86611-87114	F
TRB0V11-1	Scf.RV11-1	direct	NW_025550634.1:85142-85736	F
TRB0V11-2	Scf.RV11-2_P	direct	NW_025550634.1:80528-81070	P
TRB0V3-3	Scf.RV3-3	direct	NW_025550634.1:75509-76013	F
TRB0V11-3	Scf.RV11-3	direct	NW_025550634.1:68501-69087	F
TRB0V3-4	Scf.RV3-4	direct	NW_025550634.1:58241-58745	F
TRB0V10-3	Scf.RV10-3	direct	NW_025550634.1:51593-52093	F
TRB0V3-5	Scf.RV3-5	direct	NW_025550634.1:50546-51049	F
TRB0V11-4	Scf.RV11-4_F	direct	NW_025550634.1:48060-48873	F
TRB0V11-5	Scf.RV11-5_P	direct	NW_025550634.1:42705-43298	P
TRB0V11-6	Scf.RV11-6_P	direct	NW_025550634.1:36070-36608	P
TRB0V3-6	Scf.RV3-6	direct	NW_025550634.1:31062-31566	F
TRB0V11-7	Scf.RV11-7	direct	NW_025550634.1:24010-24598	F
TRB0V11-8	Scf.RV11-8_F	direct	NW_025550634.1:19137-19678	F*
TRB0V3-7	Scf.RV3-7	direct	NW_025550634.1:14122-14626	F
TRB0V11-9	Scf.RV11-9_F	direct	NW_025550634.1:4562-5101	F

***Oncorhynchus mykiss* TRB loci sequences (annotation of Arlee genome assembly: GCA_013265735.3)**

NC_048589.1 (Chr25) containing for the TRB1 locus a total of 51 genes: 39 TRB1V genes (35 F, 4 P), 1 TRBD, 10 TRB1J and 1 TRBC genes, and for the TRB2 locus a total of 142 genes : 130 TRB2V genes (113 F, 8 ORF, 9 P), 1 TRB2D, 10 TRB2J and 1 TRB2C genes.

NC_048583.1 (Chr19) containing for the TRB3 locus a total of 25 genes: 19 TRB1V genes (13 F, 4 ORF, 2 P), 1 TRBD, 4 TRB1J and 1 TRBC genes

***Oncorhynchus mykiss* (rainbow trout) Arlee T cell receptor beta (TRB) genes on chr. 25**
GCA_013265735.3 : TRB1 (TRB25R) and TRB2 (TRB25), NC_048589.1

**TRB1 (REV)**				
**IMGT-NC gene name**	**Gene name**	**Gene orientation in locus**	**Genomic coordinates on *Oncorhynchus mykiss* chr. 25**	**Functionality**
TRB1V6-2	TRB25RV6-2-P	direct	**NC_048589.1:43415581-43416081**	**P**
TRB1V2-1	TRB25RV2-1-F	direct	**NC_048589.1:43366935-43367423**	**F**
TRB1V3-1	TRB25RV3-1-F	direct	**NC_048589.1:43364404-43364865**	**F**
TRB1V2-2	TRB25RV2-2-F	direct	**NC_048589.1:43360982-43361665**	**F**
TRB1V1-1	TRB25RV1-1-F	direct	**NC_048589.1:43356678-43357216**	**F**
TRB1V13-1	TRB25RV13-1-F	direct	**NC_048589.1:43352714-43353268**	**F**
TRB1V3-2	TRB25RV3-2-F	direct	**NC_048589.1:43351273-43351734**	**F**
TRB1V2-3	TRB25RV2-3-F	direct	**NC_048589.1:43348177-43348732**	**F**
TRB1V1-2	TRB25RV1-2-F	direct	**NC_048589.1:43343450-43343757**	**F**
TRB1V13-2	TRB25RV13-2-F	direct	**NC_048589.1:43341578-43342079**	**F**
TRB1V3-3	TRB25RV3-3-F	direct	**NC_048589.1:43340273-43340734**	**F**
TRB1V2-4	TRB25RV2-4-P	direct	**NC_048589.1:43336765-43337269**	**P**
TRB1V1-3	TRB25RV1-3-F	direct	**NC_048589.1:43333082-43333613**	**F**
TRB1V3-4	TRB25RV3-4-F	direct	**NC_048589.1:43330950-43331411**	**F**
TRB1V2-5	TRB25RV2-5-F	direct	**NC_048589.1:43327858-43328364**	**F**
TRB1V3-5	TRB25RV3-5-F	direct	**NC_048589.1:43325422-43325915**	**F**
TRB1V2-6	TRB25RV2-6-F	direct	**NC_048589.1:43322369-43322878**	**F**
TRB1V1-4	TRB25RV1-4-F	direct	**NC_048589.1:43318210-43318748**	**F**
TRB1V13-3	TRB25RV13-3-F	direct	**NC_048589.1:43316645-43317169**	**F**
TRB1V3-6	TRB25RV3-6-F	direct	**NC_048589.1:43313899-43314376**	**F**
TRB1V2-7	TRB25RV2-7-F	direct	**NC_048589.1:43309908-43310457**	**F**
TRB1V5-1	TRB25RV5-1-F	direct	**NC_048589.1:43308255-43308961**	**F**
TRB1V5-2	TRB25RV5-2-F	direct	**NC_048589.1:43307430-43307924**	**F**
TRB1V4-1	TRB25RV4-1-F	direct	**NC_048589.1:43306638-43307157**	**F**
TRB1V5-3	TRB25RV5-3-F	direct	**NC_048589.1:43299134-43299605**	**F**
TRB1V4-2	TRB25RV4-2-F	direct	**NC_048589.1:43298345-43298861**	**F**
TRB1V5-4	TRB25RV5-4-F	direct	**NC_048589.1:43294068-43294539**	**F**
TRB1V4-3	TRB25RV4-3-F	direct	**NC_048589.1:43293279-43293795**	**F**
TRB1V5-5	TRB25RV5-5-F	direct	**NC_048589.1:43289018-43289489**	**F**
TRB1V4-4	TRB25RV4-4-F	direct	**NC_048589.1:43288229-43288745**	**F**
TRB1V5-6	TRB25RV5-6-F	direct	**NC_048589.1:43282837-43283308**	**F**
TRB1V12-1	TRB25RV12-1-F	direct	**NC_048589.1:43274129-43274530**	**F**

TRB1V2-8	TRB25RV2-8-F	direct	**NC_048589.1:43262684-43263254**	**F**
TRB1V5-7	TRB25RV5-7-F	direct	**NC_048589.1:43260171-43260665**	**F**
TRB1V4-5	TRB25RV4-5-F	direct	**NC_048589.1:43259391-43259900**	**F**
TRB1V8-1	TRB25RV8-1-F	direct	**NC_048589.1:43236610-43237105**	**F**
TRB1V6-1	TRB25RV6-1-F	direct	**NC_048589.1:43233493-43235747**	**F**
TRB1V7-1	TRB25RV7-1-P	direct	**NC_048589.1:43221010-43221399**	**P**
TRB1V7-2	TRB25RV7-2-P	direct	**NC_048589.1:43215479-43215876**	**P**
TRB1D	TRB25RD	direct	**NC_048589.1:43181850-43181928**	**F**
TRB1J1	TRB25RJ1	direct	**NC_048589.1:43181422-43181500**	**F**
TRB1J2	TRB25RJ2	direct	**NC_048589.1:43181262-43181341**	**F**
TRB1J3	TRB25RJ3	direct	**NC_048589.1:43181033-43181111**	**F**
TRB1J4	TRB25RJ4	direct	**NC_048589.1:43180827-43180907**	**F**
TRB1J5	TRB25RJ5	direct	**NC_048589.1:43180458-43180539**	**F**
TRB1J6	TRB25RJ6	direct	**NC_048589.1:43180222-43180303**	**F**
TRB1J7	TRB25RJ7	direct	**NC_048589.1:43179825-43179902**	**F**
TRB1J8	TRB25RJ8	direct	**NC_048589.1:43178877-43178953**	**F**
TRB1J9	TRB25RJ9	direct	**NC_048589.1:43178532-43178609**	**F**
TRB1J10	TRB25RJ10	direct	**NC_048589.1:43178162-43178242**	**F**
TRB1C	TRB25RC	direct	**NC_048589.1:43172506-43177117**	**F**

**TRB2 (FWD)**				
**IMGT-NC gene name**	**Gene name**	**Gene orientation in locus**	**Genomic coordinates on *Oncorhynchus mykiss* chr. 25**	**Functionality**
TRB2V2-1	TRB25V2-1-P	direct	**NC_048589.1:43463614-43464056**	P
TRB2V1-1	TRB25V1-1-P	direct	**NC_048589.1:43467674-43468209**	P
TRB2V13-1	TRB25V13-1-F	direct	**NC_048589.1:43470005-43470521**	F
TRB2V3-1	TRB25V3-1-F	direct	**NC_048589.1:43471567-43472028**	F
TRB2V2-2	TRB25V2-2-F	direct	**NC_048589.1:43475984-43476469**	F
TRB2V13-2	TRB25V13-2-F	direct	**NC_048589.1:43477999-43478598**	F
TRB2V3-2	TRB25V3-2-P	direct	**NC_048589.1:43479319-43479773**	P
TRB2V2-3	TRB25V2-3-F	direct	**NC_048589.1:43482997-43483477**	F
TRB2V1-2	TRB25V1-2-F	direct	**NC_048589.1:43486345-43486880**	F
TRB2V13-3	TRB25V13-3-F	direct	**NC_048589.1:43489517-43490041**	F
TRB2V3-3	TRB25V3-3-F	direct	**NC_048589.1:43490433-43490894**	F
TRB2V2-4	TRB25V2-4-F	direct	**NC_048589.1:43493866-43494372**	F
TRB2V3-4	TRB25V3-4-F	direct	**NC_048589.1:43497801-43498262**	F
TRB2V2-5	TRB25V2-5-F	direct	**NC_048589.1:43501331-43501808**	F
TRB2V1-3	TRB25V1-3-F	direct	**NC_048589.1:43505107-43505642**	F
TRB2V13-4	TRB25V13-4-F	direct	**NC_048589.1:43507232-43507748**	F
TRB2V3-5	TRB25V3-5-F	direct	**NC_048589.1:43508359-43508820**	F
TRB2V10-1	TRB25V10-1-ORF	direct	**NC_048589.1:43524437-43524894**	ORF
TRB2V3-6	TRB25V3-6-F	direct	**NC_048589.1:43525626-43526087**	F

TRB2V2-6	TRB25V2-6-F	direct	**NC_048589.1:43528638-43529359**	F
TRB2V3-7	TRB25V3-7-F	direct	**NC_048589.1:43532596-43533057**	F
TRB2V2-7	TRB25V2-7-F	direct	**NC_048589.1:43536076-43536545**	F
TRB2V1-4	TRB25V1-4-F	direct	**NC_048589.1:43539522-43540060**	F
TRB2V13-5	TRB25V13-5-F	direct	**NC_048589.1:43540942-43541443**	F
TRB2V3-8	TRB25V3-8-F	direct	**NC_048589.1:43541978-43542439**	F
TRB2V2-8	TRB25V2-8-F	direct	**NC_048589.1:43548358-43548868**	F
TRB2V1-5	TRB25V1-5-F	direct	**NC_048589.1:43554949-43555480**	F
TRB2V13-6	TRB25V13-6-F	direct	**NC_048589.1:43556575-43557091**	F
TRB2V3-9	TRB25V3-9-F	direct	**NC_048589.1:43557841-43558302**	F
TRB2V2-9	TRB25V2-9-F	direct	**NC_048589.1:43562958-43563420**	F
TRB2V1-6	TRB25V1-6-F	direct	**NC_048589.1:43566860-43567398**	F
TRB2V3-10	TRB25V3-10-F	direct	**NC_048589.1:43570662-43571123**	F
TRB2V2-10	TRB25V2-10-F	direct	**NC_048589.1:43574153-43574664**	F
TRB2V1-7	TRB25V1-7-F	direct	**NC_048589.1:43578444-43578979**	F
TRB2V3-11	TRB25V3-11-F	direct	**NC_048589.1:43581317-43581778**	F
TRB2V2-11	TRB25V2-11-F	direct	**NC_048589.1:43584328-43584846**	F
TRB2V1-8	TRB25V1-8-F	direct	**NC_048589.1:43590912-43591457**	F
TRB2V13-7	TRB25V13-7-F	direct	**NC_048589.1:43592159-43592660**	F
TRB2V3-12	TRB25V3-12-F	direct	**NC_048589.1:43593504-43593965**	F
TRB2V2-12	TRB25V2-12-F	direct	**NC_048589.1:43596994-43597505**	F
TRB2V1-9	TRB25V1-9-P	direct	**NC_048589.1:43601177-43601711**	P
TRB2V2-13	TRB25V2-13-F	direct	**NC_048589.1:43607508-43608557**	F
TRB2V1-10	TRB25V1-10-F	direct	**NC_048589.1:43610911-43611449**	F
TRB2V3-13	TRB25V3-13-F	direct	**NC_048589.1:43613571-43614032**	F
TRB2V3-14	TRB25V3-14-F	direct	**NC_048589.1:43625254-43625715**	F
TRB2V2-14	TRB25V2-14-F	direct	**NC_048589.1:43628099-43628807**	F
TRB2V1-11	TRB25V1-11-F	direct	**NC_048589.1:43632000-43632538**	F
TRB2V13-8	TRB25V13-8-F	direct	**NC_048589.1:43633908-43634430**	F
TRB2V3-15	TRB25V3-15-F	direct	**NC_048589.1:43635040-43635501**	F
TRB2V2-15	TRB25V2-15-F	direct	**NC_048589.1:43638985-43639531**	F
TRB2V1-12	TRB25V1-12-F	direct	**NC_048589.1:43646538-43647073**	F
TRB2V13-9	TRB25V13-9-F	direct	**NC_048589.1:43648938-43649460**	F
TRB2V3-16	TRB25V3-16-F	direct	**NC_048589.1:43650070-43650532**	F
TRB2V1-13	TRB25V1-13-F	direct	**NC_048589.1:43657872-43658407**	F
TRB2V13-10	TRB25V13-10-F	direct	**NC_048589.1:43660758-43661273**	F
TRB2V3-17	TRB25V3-17-F	direct	**NC_048589.1:43662421-43662882**	F
TRB2V1-14	TRB25V1-14-F	direct	**NC_048589.1:43671721-43672259**	F
TRB2V13-11	TRB25V13-11-F	direct	**NC_048589.1:43675887-43676411**	F
TRB2V3-18	TRB25V3-18-F	direct	**NC_048589.1:43677022-43677483**	F
TRB2V2-16	TRB25V2-16-F	direct	**NC_048589.1:43679639-43680276**	F
TRB2V1-15	TRB25V1-15-F	direct	**NC_048589.1:43683589-43684127**	F
TRB2V13-12	TRB25V13-12-ORF	direct	**NC_048589.1:43686152-43686704**	ORF
TRB2V3-19	TRB25V3-19-F	direct	**NC_048589.1:43687748-43688209**	F
TRB2V2-17	TRB25V2-17-ORF	direct	**NC_048589.1:43691299-43691804**	ORF

TRB2V3-20	TRB25V3-20-F	direct	**NC_048589.1:43694012-43694473**	F
TRB2V2-18	TRB25V2-18-F	direct	**NC_048589.1:43708466-43708948**	F
TRB2V1-16	TRB25V1-16-F	direct	**NC_048589.1:43716365-43716900**	F
TRB2V3-21	TRB25V3-21-F	direct	**NC_048589.1:43718661-43719123**	F
TRB2V2-19	TRB25V2-19-F	direct	**NC_048589.1:43721879-43722408**	F
TRB2V1-17	TRB25V1-17-ORF	direct	**NC_048589.1:43728278-43728714**	ORF
TRB2V13-13	TRB25V13-13-ORF	direct	**NC_048589.1:43731776-43732386**	ORF
TRB2V3-22	TRB25V3-22-F	direct	**NC_048589.1:43733139-43733600**	F
TRB2V2-20	TRB25V2-20-F	direct	**NC_048589.1:43736524-43737042**	F
TRB2V1-18	TRB25V1-18-F	direct	**NC_048589.1:43744425-43744970**	F
TRB2V13-14	TRB25V13-14-F	direct	**NC_048589.1:43747003-43747567**	F
TRB2V3-23	TRB25V3-23-F	direct	**NC_048589.1:43748177-43748638**	F
TRB2V2-21	TRB25V2-21-F	direct	**NC_048589.1:43752328-43752810**	F
TRB2V3-24	TRB25V3-24-F	direct	**NC_048589.1:43755028-43755489**	F
TRB2V2-22	TRB25V2-22-F	direct	**NC_048589.1:43758267-43758854**	F
TRB2V1-19	TRB25V1-19-F	direct	**NC_048589.1:43765269-43765666**	F
TRB2V13-15	TRB25V13-15-F	direct	**NC_048589.1:43766657-43767181**	F
TRB2V3-25	TRB25V3-25-F	direct	**NC_048589.1:43767791-43768237**	F
TRB2V2-23	TRB25V2-23-F	direct	**NC_048589.1:43771223-43771714**	F
TRB2V1-20	TRB25V1-20-ORF	direct	**NC_048589.1:43784442-43784977**	ORF
TRB2V13-16	TRB25V13-16-F	direct	**NC_048589.1:43786180-43786681**	F
TRB2V3-26	TRB25V3-26-F	direct	**NC_048589.1:43787529-43787990**	F
TRB2V2-24	TRB25V2-24-F	direct	**NC_048589.1:43791019-43791529**	F
TRB2V1-21	TRB25V1-21-P	direct	**NC_048589.1:43795297-43795837**	P
TRB2V13-17	TRB25V13-17-F	direct	**NC_048589.1:43797207-43797731**	F
TRB2V3-27	TRB25V3-27-F	direct	**NC_048589.1:43798342-43798803**	F
TRB2V2-25	TRB25V2-25-F	direct	**NC_048589.1:43801353-43801869**	F
TRB2V1-22	TRB25V1-22-F	direct	**NC_048589.1:43807963-43808508**	F
TRB2V13-18	TRB25V13-18-F	direct	**NC_048589.1:43809163-43809664**	F
TRB2V3-28	TRB25V3-28-F	direct	**NC_048589.1:43810508-43810969**	F
TRB2V2-26	TRB25V2-26-F	direct	**NC_048589.1:43812103-43812822**	F
TRB2V13-19	TRB25V13-19-F	direct	**NC_048589.1:43816215-43816751**	F
TRB2V3-29	TRB25V3-29-F	direct	**NC_048589.1:43817362-43817823**	F
TRB2V2-27	TRB25V2-27-F	direct	**NC_048589.1:43820853-43821360**	F
TRB2V1-23	TRB25V1-23-F	direct	**NC_048589.1:43824373-43824871**	F
TRB2V13-20	TRB25V13-20-F	direct	**NC_048589.1:43825912-43826428**	F
TRB2V3-30	TRB25V3-30-F	direct	**NC_048589.1:43827039-43827500**	F
TRB2V10-2	TRB25V10-2-ORF	direct	**NC_048589.1:43842023-43842484**	ORF
TRB2V3-31	TRB25V3-31-F	direct	**NC_048589.1:43843075-43843536**	F
TRB2V2-28	TRB25V2-28-F	direct	**NC_048589.1:43846087-43846830**	F
TRB2V1-24	TRB25V1-24-F	direct	**NC_048589.1:43855012-43855547**	F
TRB2V2-29	TRB25V2-29-F	direct	**NC_048589.1:43860450-43861613**	F
TRB2V1-25	TRB25V1-25-F	direct	**NC_048589.1:43864896-43865431**	F
TRB2V2-30	TRB25V2-30-F	direct	**NC_048589.1:43870720-43871848**	F
TRB2V1-26	TRB25V1-26-P	direct	**NC_048589.1:43875137-43875675**	P

TRB2V13-21	TRB25V13-21-F	direct	**NC_048589.1:43877153-43877677**	F
TRB2V3-32	TRB25V3-32-F	direct	**NC_048589.1:43879137-43879598**	F
TRB2V2-31	TRB25V2-31-P	direct	**NC_048589.1:43883288-43883782**	P
TRB2V3-33	TRB25V3-33-F	direct	**NC_048589.1:43885841-43886302**	F
TRB2V2-32	TRB25V2-32-F	direct	**NC_048589.1:43890245-43890714**	F
TRB2V1-27	TRB25V1-27-F	direct	**NC_048589.1:43894029-43894564**	F
TRB2V13-22	TRB25V13-22-F	direct	**NC_048589.1:43895275-43895778**	F
TRB2V1-28	TRB25V1-28-F	direct	**NC_048589.1:43899806-43900344**	F
TRB2V3-34	TRB25V3-34-F	direct	**NC_048589.1:43907276-43907737**	F
TRB2V2-33	TRB25V2-33-F	direct	**NC_048589.1:43910108-43910560**	F
TRB2V1-29	TRB25V1-29-F	direct	**NC_048589.1:43916960-43917491**	F
TRB2V3-35	TRB25V3-35-P	direct	**NC_048589.1:43919373-43919834**	P
TRB2V2-34	TRB25V2-34-F	direct	**NC_048589.1:43922384-43922890**	F
TRB2V1-30	TRB25V1-30-F	direct	**NC_048589.1:43928895-43929430**	F
TRB2V13-23	TRB25V13-23-P	direct	**NC_048589.1:43930697-43931221**	P
TRB2V3-36	TRB25V3-36-F	direct	**NC_048589.1:43931831-43932293**	F
TRB2V2-35	TRB25V2-35-F	direct	**NC_048589.1:43935057-43935562**	F
TRB2V5-1	TRB25V5-1-F	direct	**NC_048589.1:43939918-43940412**	F
TRB2V5-2	TRB25V5-2-F	direct	**NC_048589.1:43946063-43946535**	F
TRB2V9-1	TRB25V9-1-F	direct	**NC_048589.1:43960167-43960850**	F
TRB2V7-1	TRB25V7-1-ORF	direct	**NC_048589.1:43966196-43966581**	ORF
TRB2D	TRB25D-F	direct	**NC_048589.1:43974664-43974742**	F
TRB2J1	TRB25J1_F	direct	**NC_048589.1:43975091-43975169**	F
TRB2J2	TRB25J2_ORF	direct	**NC_048589.1:43975250-43975329**	ORF
TRB2J3	TRB25J3_F	direct	**NC_048589.1:43975480-43975558**	F
TRB2J4	TRB25J4_F	direct	**NC_048589.1:43975691-43975771**	F
TRB2J5	TRB25J5_F	direct	**NC_048589.1:43976059-43976140**	F
TRB2J6	TRB25J6_F	direct	**NC_048589.1:43976295-43976376**	F
TRB2J7	TRB25J7_F	direct	**NC_048589.1:43976702-43976779**	F
TRB2J8	TRB25J8_F	direct	**NC_048589.1:43977382-43977458**	F
TRB2J9	TRB25J9_F	direct	**NC_048589.1:43977726-43977803**	F
TRB2J10	TRB25J10_F	direct	**NC_048589.1:43978085-43978165**	F
TRB2C	TRB25C-F	direct	**NC_048589.1:43979152-43984073**	F

***Oncorhynchus mykiss* (rainbow trout) Arlee T cell receptor beta (TRB) genes on chr. 19**
GCA_013265735.3 : TRB3 (TRB19R), NC_048583.1

**IMGT-NC gene names**	**Gene names**	**Gene orientation in locus**	**Genomic coordinates on *Oncorhynchus mykiss* chr. 19**	**Functionality**
TRB3V7-1	TRB19RV7-1-F	opposite	**NC_048583.1:64276391-64276842**	F
TRB3V5-1	TRB19RV5-1-P	opposite	**NC_048583.1:64274020-64274505**	P
TRB3V10-1	TRB19RV10-1-ORF	direct	**NC_048583.1:63879281-63878788**	ORF
TRB3V3-1	TRB19RV3-1-F	direct	**NC_048583.1:63878148-63877684**	F
TRB3V11-1	TRB19RV11-1-F	direct	**NC_048583.1:63872293-63871762**	F
TRB3V10-2	TRB19RV10-2-ORF	direct	**NC_048583.1:63863185-63862725**	ORF
TRB3V3-2	TRB19RV3-2-F	direct	**NC_048583.1:63862085-63861621**	F
TRB3V11-2	TRB19RV11-2-F	direct	**NC_048583.1:63855096-63854565**	F
TRB3V10-3	TRB19RV10-3-ORF	direct	**NC_048583.1:63846015-63845555**	ORF
TRB3V3-3	TRB19RV3-3-F	direct	**NC_048583.1:63844915-63844451**	F
TRB3V11-3	TRB19RV11-3-F	direct	**NC_048583.1:63837926-63837397**	F
TRB3V10-4	TRB19RV10-4-ORF	direct	**NC_048583.1:63829114-63828654**	ORF
TRB3V3-4	TRB19RV3-4-F	direct	**NC_048583.1:63828014-63827550**	F
TRB3V11-4	TRB19RV11-4-F	direct	**NC_048583.1:63821025-63820496**	F
TRB3V10-5	TRB19RV10-5-F	direct	**NC_048583.1:63812171-63811711**	F
TRB3V3-5	TRB19RV3-5-F	direct	**NC_048583.1:63811071-63810607**	F
TRB3V4-1	TRB19RV4-1-P	direct	**NC_048583.1:63800657-63800237**	P
TRB3V5-2	TRB19RV5-2-F	direct	**NC_048583.1:63799860-63799361**	F
TRB3V4-2	TRB19RV4-2-F	direct	**NC_048583.1:63785914-63785404**	F
TRB3D	TRB19RD	direct	**NC_048583.1:63769887-63769965**	F
TRB3J1	TRB19RJ1_F	direct	**NC_048583.1:63766993-63767071**	F
TRB3J2	TRB19RJ2_F	direct	**NC_048583.1:63766816-63766896**	F
TRB3J3	TRB19RJ3_ORF	direct	**NC_048583.1:63766593-63766682**	ORF
TRB3J4	TRB19RJ4_F	direct	**NC_048583.1:63766376-63766459**	F
TRB3C	TRB19RC	direct	**NC_048583.1:63760837-63763852**	F
