# Supplementary material for: Evolution of T cell receptor beta loci in salmonids
Source: Front Immunol. 2023 Aug 15;14:1238321. doi: 10.3389/fimmu.2023.1238321 (PMC10464911; doi:10.3389/fimmu.2023.1238321)
Supplement: Supplementary file 2 [file DataSheet_2.pdf]

Supplementary File 2: Organization of TRB loci in rainbow trout (*Onchorynchus mykiss*, Arlee strain).

A) TRB loci located on chromosome 25, **B)** on chromosome 19. TRBV gene names are according to IMGT nomenclature. Functional TRBV genes are in red. TRBV genes with an open reading frame, and pseudogenes with frameshift(s) in the V-REGION or without -RS, are in dark and light grey, respectively. The arrow indicates the transcriptional orientation. The symbols representing the genes are not to scale. **C)** and **D)** detailed view of the TRBC-D-J cluster located on chromosome 25 and 19, respectively. The boxes representing the genes are not to scale.

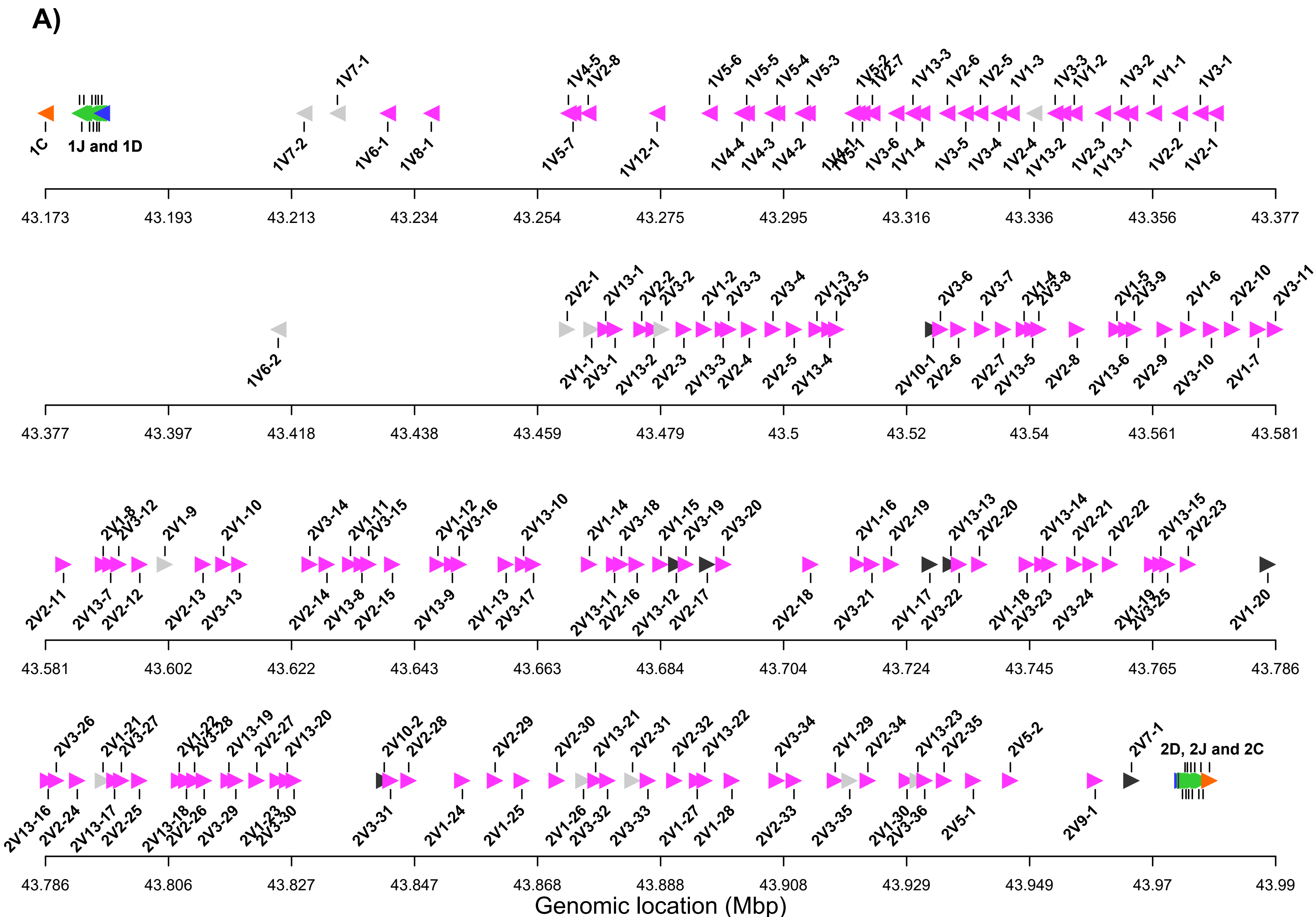
A)
1C
1J and 1D
2D, 2J and 2C
Genomic location (Mbp)
43.173
43.193
43.213
43.234
43.254
43.275
43.295
43.316
43.336
43.356
43.377
43.397
43.418
43.438
43.459
43.479
43.5
43.52
43.54
43.561
43.581
43.602
43.622
43.643
43.663
43.684
43.704
43.724
43.745
43.765
43.786
43.806
43.827
43.847
43.868
43.888
43.908
43.929
43.949
43.97
43.99

B)

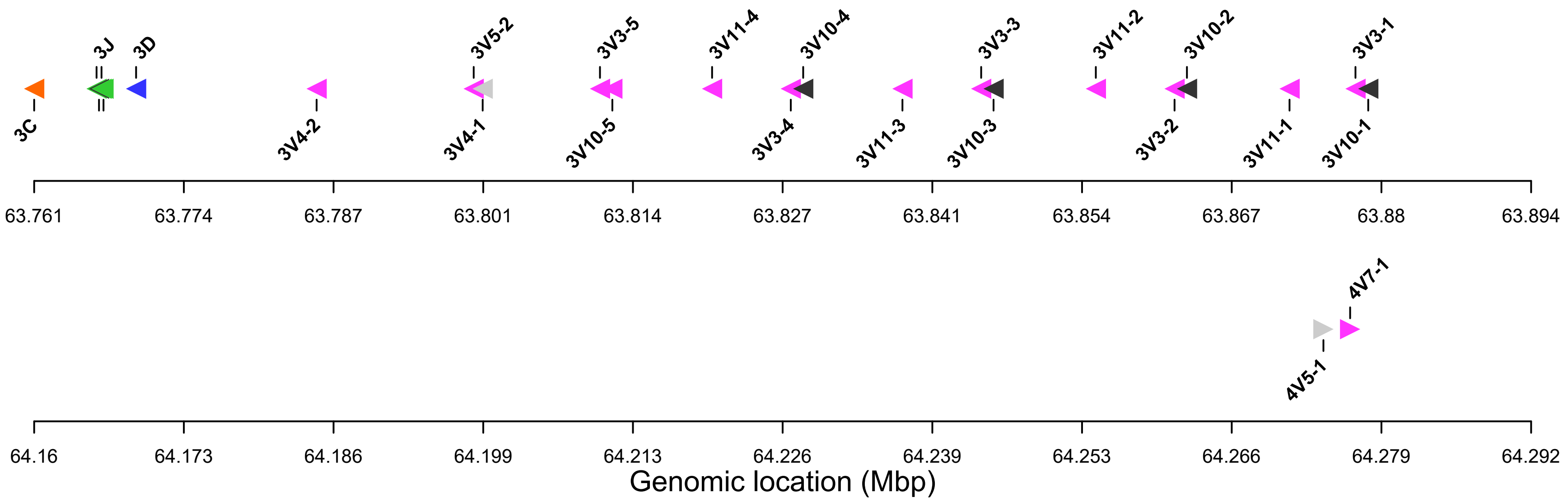
3J
3D
3V5-2
3V3-5
3V11-4
3V10-4
3V3-3
3V11-2
3V10-2
3V3-1
3C
3V4-2
3V4-1
3V10-5
3V3-4
3V11-3
3V10-3
3V3-2
3V11-1
3V10-1
63.761
63.774
63.787
63.801
63.814
63.827
63.841
63.854
63.867
63.88
63.894
4V7-1
4V5-1
64.16
64.173
64.186
64.199
64.213
64.226
64.239
64.253
64.266
64.279
64.292
Genomic location (Mbp)

C)

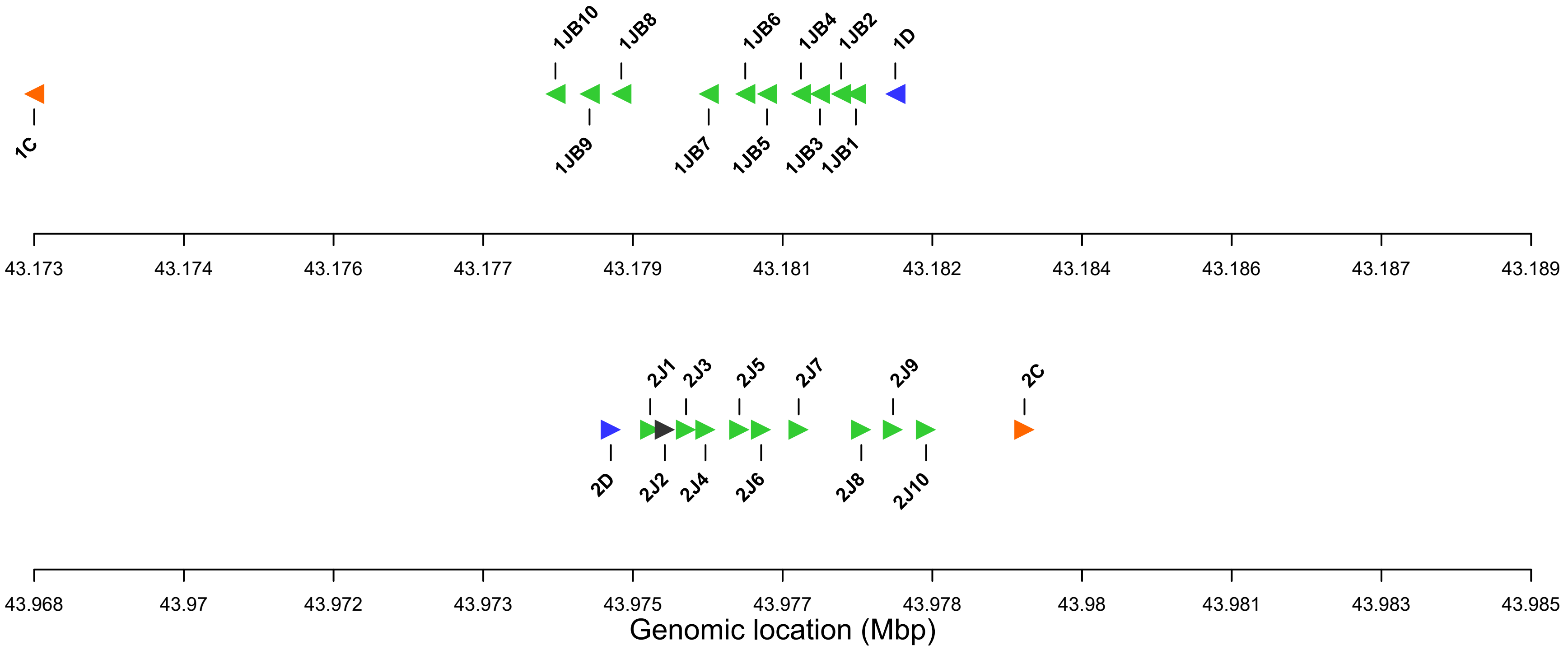
1JB10
1JB8
1JB6
1JB4
1JB2
1D
1C
1JB9
1JB7
1JB5
1JB3
1JB1
43.173
43.174
43.176
43.177
43.179
43.181
43.182
43.184
43.186
43.187
43.189
2J1
2J3
2J5
2J7
2J9
2C
2D
2J2
2J4
2J6
2J8
2J10
43.968
43.97
43.972
43.973
43.975
43.977
43.978
43.98
43.981
43.983
43.985
Genomic location (Mbp)

D)

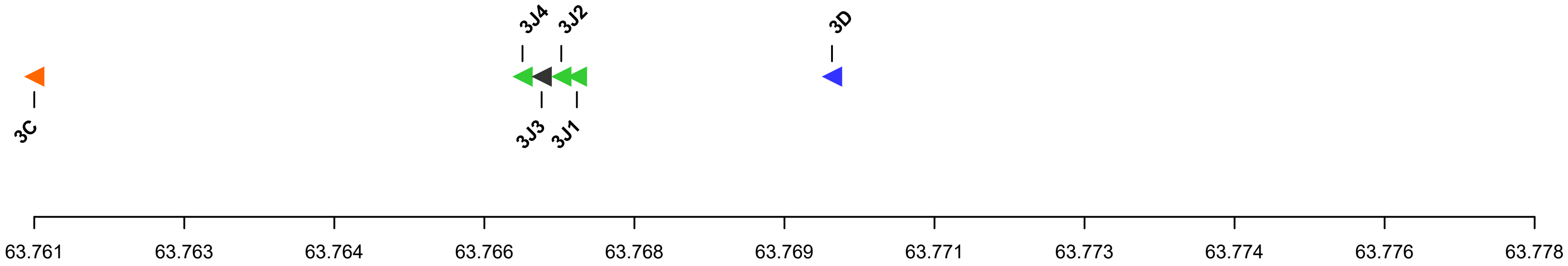
3J4
3J2
3D
3C
3J3
3J1
63.761
63.763
63.764
63.766
63.768
63.769
63.771
63.773
63.774
63.776
63.778
