# Supplementary material for: Evolution of T cell receptor beta loci in salmonids
Source: Front Immunol. 2023 Aug 15;14:1238321. doi: 10.3389/fimmu.2023.1238321 (PMC10464911; doi:10.3389/fimmu.2023.1238321)
Supplement: Supplementary file 3 [file DataSheet_3.pdf]

Supplementary File 3. Genomic localization of the 4 exons and RS sequences belonging to functional TRBV6 gene.

Nucleotide sequences of the GENE-UNIT (from start codon ATG to V-RS included) of the TRB1V6 gene in the 5 salmonid species: rainbow trout, Arlee strain (aOncmyk); Atlantic salmon (Salsal), Chinook salmon (Onctsh), Chum salmon (Oncket), Pink salmon (Oncgor). For Oncmyk (Arlee strain), only the sequence of the functional gene TRB1V6-1 is shown.

The leader region (exon L-PART1 and L-PART2) is in yellow. The 3 exons encoding the V-REGION are in green. The V-RS is in pink (V_HEPTAMER and V_NONAMER) and in grey (spacer).

> NC_048589.1 – aOncmyk_TRB1V6-1_ USDA_OmykA_1.1 – Chr25- 43235747-43233454

ATGACGCATCCTATAGTCCTCCTGAGTTGGCTGTTTCTCCTGTCCCCAAGTGAGTGTCTGAGGTTAGCTTTG
TAGCTGGGATTCTTGGAGTAATTAGAGTTGTACCAACTCTTAATAGACACAGTGATGGCTTGTCTCTCTTCT
TCCTCCTCTCTTCTCCTCTCTTCCTCTCCTGTCCCCTGATCCTCTCCCCTGCCCTGCTCTCCTCTCTTCTCAAC
AGGTCAGTGTCGAGCAGCAGGCCCCGTAGTGCTTCAGCCCAGACAGCTCCTCACCCTTCCTGGTAACACC
ACCACCTCCAGTCCAGTCACCCTGCAGTGCTCCCTGGGTCCAGGCTTCAGCATGCCCAGCTACACCATGAT
GTGGTACCGACAGGTCAGAGAAGTTACCAGGGCATGACACCATGATGTGGTACTGATAGGTCAGAGAAG
TTACCAGGGCATTACACCATGATGTGGTACTGATAGGTCAGAGAAGTTACCAGGGCATTACACCATGATGT
GGTACCGACAGGTCAGAGAAGTTACCAGGGCATTACACCATGTGGCCAGCAGGTTGGCATAGGCTGATC
TGATACATCAGCCCTCCCTAATTCATCAGACCAGCCTAGTTCATCAGCCCTCCCTAATTCATCAGACCAGCC
TAATTCATCAGACCAGCCTAATGCATCAGACCAGCCTAGTTCATCAGCCCTCCCTAATTCATCAGACCAGCC
TAATTCATCAGACCAGCCTAATTCATCAGACCAGCCTAATGCATCAGACCAGCCTAATTCATCAGCCCTCCC
TAATTCACCCTCCTGTCTCAGCCTCCAGTATTTATGCTGCAGTAGTTTATGCGTCAGGGGGCTAGGGTCAGT
TTGTTATATCTGGAGTACTTCTCCTGTCGTATCCGGTGTCCTGTGTGAATTTGGGGTGGAGTGGAGTGGCC
AGCAGGTTGCTAGGTGAGGTGGGGTGGAGTGGCCAGCAGGTTGCTAGGTGAGGTGGGGTGGAGTGGA
GTGGCCAGCAGGTTGGTAAGTGAGGTGGAGTGGGGTGGGGTGGAGTGGCCAGCAGGTTGGTAGGTGA
GGTGGGGTGGCCAGCAGGTTGGTAGGTGAGGTGGAGTGGGGTGGGGTGGAGTGGCCAGCAGGTTGG
TAGGTGAGGTGGGGTGGCCAGCAGGTTGGTAGGTGGGGTGGGGTGGGGTGGCCAGCAGGTTGGTAG
GTGGGGTGGAGTGGCCAGCAGGTTGGTAGGTGAGGTGGGGTGTCCAGCAGATTCGTAGGTGGGGTGG
CCAGCAGGTTGGTAGGTGAGGTGGGGTGTCCAGCAGATTCGTAGGTGGGGTGGCCAGCAGGTTGGTAG
GTGAGGTGGGGTGGGGTGGGGTGGAGTGGCCAGCAGGTTGGTAGGTGAGGTGGGGTGGCCAGCAGG
TTGGTAGGTGGGGTGGGGTGGGGTGGCCAGCAGGTTGGTAGGTGGGGTGGAGTGGGGTGGGGTGGG
GTGGCCAAAAGGTTGGTAAGTGAGGTGGAGTGGGGTGGGGTGGAGTGGCCAGCAGGTTGGTAGGTGA
GGTGGGGTGGCCAGCAGGTTGGTAGGTGGGGTGGGGTGGGGTGGCCAGCAGGTTGGTAGGTGGGGT
GGAGTGGCCAGCAGGTTGGTAGGTGAGGTGGGGTGTCCAGCAGATTCATAGGTGGGGTGGCCAGCAG
GTTGGTAGGTGAGGTGGGGTGTCCAGCAGATTCGTAGGTGGGGTGGCCAGCAGGTTGGTAGGTGAGGT
GGGGTGTCCAGCAGATTCGTAGGTGGGGTGGCCAGCAGGTTGGTAGGTGAGGTGGGGTGTCCAGCAG
ATTCGTAGGTGGGGTGGCCAGCAGGTTGGTAGGTGAGGTACCATAACATGCTTTGGTACCAACAGGTCC
GCTATGGAGGTCCAGTGGAGTTCCTCATTAAGGAGTACGAGAAAACCAAGGGGCGATACAAAGAGGAC
CTGGACACATCGGGGAACAGGTTCATCCTGCAGGTGATGGAGAGCATCTGAGCCGGGTTCCTCTTGACG
TTTCTTCTCTCTGTGTAGAGGAGAGGAGAGGAGAGGAGAGGAGAGGAGAGGAGAGGAGAGGAGAGG
AGAGGAGAGGAGAGGAGAGGAGAGGAGATGTTTCTTCTCTCTGGGTCTCGGCTGAGTTTATTTACCAGC

ACGTGCCGAATAAAAAGGCCTTTTTCTAAATGATAATTGATTGATTGATGTGTTTTGATTGGTTGCAGATTT
CTGAGCTGGTTGTGGAGGACAGTGGTGTCTACTACTGCGCTGCTAGTCACGGTGAAACAACAGCCTGAA
GAGCAGTACAAATACAA

> NC_059450.1_SalsalTRB1V6-1_ Ssal_v3.1- ssa09-47926085-47928593

ATGCATCCTATAGTCCTCCTGAGTTGGCTGTTTCTCCTGTCCCCAGGTGAGTGTCTGAGGTTAGCTTAGTAG
CTGGGATTCATGGAGTAATTAGAGTTGTACCAACTCTTAATCGACACAGTGATTGCTTGTCTCTCTTCTTCC
TCCTCTCCTCGCCTCTCTTCTCCTCTCTTCCTCTCCCCTGCCCTCCTCTCCTCTCTTCTCTACAGGTCAATGTG
GAGCAGCAGACCCCGTAGTGCTTCAGCCCAGACAGCTCCTCACCCTTCCTGGTAACACCACCACCTCCGG
TCCAGTCACTCTGCAGTGCTCCCTGGGTCCAGGCTTCAGCATGCCCAGCTACACAATGATGTGGTACCGAC
AGGTCAGAGAAGTCACCAGGGCATTACACCATGATGTGGTACTGACAGGTCAGAGAAGTCACCAGGGCA
TTACACCATGATGTGGTACCGACAGATCAGAGAAGTCACCAGGGCATTACACCATGATGTGGTACCGACA
GGTCAGAGAAGTCACCAGGGCATTACACCATGATGTGGTACTGACAGGTCAGAGAAGTCACCAGGGCAT
TACACCATGATGTGGTACTGACAGGTCAGAGAAGTCACCAGGGCATTACACCATGATGTGGTACCGACAG
GTCAGAGAAGTGTCCAGGGCATTACACCATGATGTGGTACTGACAGGTCAGAGAAGTCACCAGGGCATT
ACACCATGATGTGGTACTGACAGGTCAGAGAAGTCACCAGGGCATTACACCATGATGTGGTACTGACAGG
TCAGAGAAGTCACCAGGGCATTATGCATGTCGGTACTCTCTTCTGTTGTAGGATCAGTGTTGAGGAGCAG
TGAACTGCATTTACTTCAACTAGTCATTTAATTACATTTTGCAGTAGCTTGGTGGTAGTTGGACTAAATTAA
AATCTTGGGAGTGTTGTCAGTAGTTAATTACTGTTTTACCATGTAGCGGTGTAGCTAACTACTGGAACTACA
CACTACTGTTTTACCATGTAGCGGTGTAGCTAACTACTGGAACTACACACTACTGTTTTACCATGTAGCGGT
GTAGTTAACTACTGGAACTACACACTACTGTTTTACCATGTTGCGGTGTAGCTAACTACTGGAACTACACAC
TACTGTTTTACCATGTAGCGGTGTAGCTAACTACTGGAACTACACACTACTGTTTTACCATGTAGCGGTGTA
GCTAACTACTGGAACTACACACTACTGATTTACCATGTAGCGGTGTAGCTAACTACTGGAACTACACACTAC
TGTTTTACCAGGTAGTGGTGTAGCTAACTACTGGACCTACACACTACTGTTTTACCATGTAGCGGTGTAGCT
AACTACTGGAACTACACACTACTGTTTTACCATGTACGGAACTACACTTTTGGAACTACACACAAATTATTT
TTTGCAGAAATTAAATACATTATATTTGAAATAGGCAAGAATTCTGCATCAGACCTGCCTAATTCATCAGAC
CTGCCTAATTCATCAGACCAGCCTAATTCATCAGACCTGCCTAATTCATCAGACCAGCCTAATTCATCAGAC
CTGCCTAATTCATCAGACCAGCCTAATTCATCAGACCTGCCTAATTCATCAGACCTGCCTAATTCATCAGACC
TGCCTAATTCATCAGACCAGCTGAATTCATCAGACCTACCTCATTCTCACTTTAAACACTTTCTGTGTGCCTG
ATTCTCTCACTTTTTGTGTTTAATTACACATTCTGTTAACATCTGACTCCAGAGTGATCTCTTCTGGCAATTT
GTAGTCTATTAAGAATAAGTATCACAGTGCTTGTGATATTGAATGTTTGGAAAGTAATATAAATCAAATCAA
ATCAAATGTATTTATAAAGCCCTTCTTACATCAGCTGATATCTCAAAGTGCTATATAGAAACCCAGCCTAAAA
CCCCAAACAGCAAGCAATGCAGGTGTAGAAGCACAGTGGCTAGGAAAAACTCCTTAGAAAGGCCAGCA
GGTTGGTAGGTGAGGTGGGGTGGCCAGCAGGTTGGTAGGTCTGGTGGGGTGAGGTGGGTTGGCCAGC
AGGTTGGTAGGTGAGGTGGGGTGGAGTGGCCAGCAGATTCGTAGGTGGGGTGGCCAACAGGTTGGTA
GGTGAGGTGGGGTGTCCAGCAGGTTAGAAGTTGTGGTACCATAACATGCTTTGGTACCAACAGGTCCGC
TATGGAGGCCCAGTGGAGTTCCTCATTAAAGAGTACGAGAAAACCCAGGGGCGATACAAAGAGAACCTG
GACACATCGGGGAACAGGTTCATCCTGCAGGTGATGGAGAGCATCTGAGCCGGGTTCCTCTTGACGTCT
CTTCTCTTTGGGTCTTGGCTGAGTTTATTTACAAGCACGTGCATAAAGGCCTTTTTCTAAATGTGTTCGATT
GACTGCAGATCTCTGAACTGGTTGTGGAGGACAGTGGGGTCTACTACTGCGCTGCTAGTCACAGTGAAAT
AACAGCTTGAAGAGCAGTACAAATACAA

>NC_056436.1- Onctsh TRB1V6- Otsh_v2.0 - LG08 -5466746-5469270

ATGACGCATCCTATAGTCCTCCTGAGTTGGCTGTTTCTCCTGTCCCCAAGTGAGTGTCTGAGGTTAGCTTTG
TAGCTGGGATTCTTGGAGTAATTAGAGTTGTACCAACTCTTAATAGACACAGTGATGGCTTGTCTCTCTTCT
TCCTCCTCTCTTCTCCTCTCTTCCTCTCCTGTCCTCTGATCCTCTCCCCTGCCCTCCTCTCCTCTCCTCTCCTCT
CTTCTCAACAGGTCAGTGTGGAGCAGCAGGCCCCATAGTGCTTCAGCCCAGACAGCTCCTCACCCTTCCT
GGTAACACCACCACCTCCAGTCCAGTCACCCTGCAGTGCTCCCTGGGTCCAGGCTTCAGCATGCCCAGCT
ACACCATGATGTGGTACCGACAGGTCAGAGAAGTTACCAGGGCATTACACCATGATGTGGTACCGACAGG
TCAGAGAATTACACCCTGATTACACCCTGATGTGGTACCGACAGGTCAGAGAAGTTACCAGGGCATTACA
CCCTGATGTGGTACCGACAGGTCAGAGAAGTTACCAGGGCATTACACCCTGATGTGGTACTGACAGGTCA
GAGAAGTTACCAGGGCATTACACCCTGATGTGGTACCGACAGGTCAGAGAAGTTACCAGGGCATTACTTC
AACTAGTCAACTAGTCATTTAATTACATTTTGCAGTAGCTTGGTGGTAGTTGAACTAAATAAAAATCTTGGT
AGTGTTTTCAGTAGTTAATTACTGTTTTACCAGGTAGCAGTGTAGCTAGCTACTGGAACTACACACTACTGT
TTTACCAGGTAGCGGTGTAGCTAGCTACTGGAACTACACACTACTGTTTTACCAGGTAGCGGTGTAGCTAG
CTACTGGAACTACACACTACTGTTTTACCAGGTAGCGGTGTGGTTAACTACTGGAACTACACACTACTGGT
TTGGGGTGGAGTGGCCAGACGGTTGGTAGGTGGGGTGGGGTGGGGTGGAGTGGAGTGGGTGGGGTG
GGGTGGGGTGGCCAGCAGGTTGGTAGGTGGGGTGGGGTGGCCAGCAGGTTGGTAGGTGAGGTGGGG
TGGAGTGGGGTGGGGTGGAGTGGCCAGCAGGTTGGTAGGTGAGGTGGGGTGGGGTGGAGTGGCCAG
CAGGTTGGTAGGTGGGGTGGGGTGGGGTGGGGTGGACAGGCCAGCAGGTTGGTAGGTGAGGTGGGG
TGGAGAGGCCAGCAGGTTGGTAGGTGGGGTGGGGTGGAGAGGCCAGCAGGTTGGTAGGTGAGCTGG
GGTGGAGAGGCCAGCAGGTTGGTAGGTGAGGTGGGGTGGGGTGGGGTGGAGAGGCCAGCAGGTTAG
TAGGTGAGGTGGGGTGGAGTGGCCAGCAGGTTGGTAGGTGAGGTGGGGTGGAGAGGCCAGCAGGTT
GGTAGGTGAGGTGGGGTGGAGTGGCCAGCAGGTTGGTAGGTGAGGTGGGGTGGAGAGGCCAGCAGG
TTGGTAGGTGAGGTGGGGTGGAGAGGCCAGCAGGTTGGTAGGTGAGGTGGGGTGGAGTGGCCAGCA
GGTTGGTAGGTGAGGTGGGGTGGAGTGGCCAGCAGGTTGGTAGGTGAGGTGGGGTGGAGAGGCCAG
CAGGTTGGTAGGTGAGCTGGGGTGGAGAGGCCAGCAGGTTGGTAGGTGAGGTGGGGTGGGGTGGGG
TGGAGAGGCCAGCAGGTTGGTAGGTGAGGTGGGGTGGAGTGGCCAGCAGGTTGGTAGGTGAGGTGG
GGTGGAGTGGCCAGCAGGTTGGTAGGTGAGGTGGCCAGCAGGTGAGGTGGGGTGTCCAGCAGGTTAG
AATGTGTGGTGGGGTGTCCAGCAGGTTAGAAGGTGTGGTGGGGTGTCCAGCAGGTTGGTAGGTGAGGT
GGGGTGTACAGCAGGTTGGTAGGTGAGGTGGGGTGTCCAGCAGGTTATAAGGTGTGGTGGGGTGTCCA
GCAGGTTGGTAGGTGAGGTGGGGTGGCCAGCGGGTTGGTAGGTGAGGTGGGGTGTCCAGCAGGTTAG
AAGGTGAGGTGGGGTGTCCAGCAGGTTAGAAGGTGTGGTACAATAACAAGCTTTGGTACCAACAGGTCC
GCTATGGAGGCCCAGTGGAGTTCCTCATTAAGGAGTACGAGAAAACCCGGGGGCGATACAAAGAGGAC
CTGGACACATCGGGGAACAGGTTCATCCTGCAGGTGATGGAGAGCATCTGAGCCGGGTTCCTCTTGACG
TTTCTTCTCTCAGTGAAGAGAAGAGGAGAGGAGAGGAGAGGAGAGGAGAGGAGAGGAGAGGAGAG
GAGAGGAGAGGAGAGGAGGAGGGAGAGGAGAGGAGAGGAGAGGAGAGGAGAGGAGAGGAGAGG
AGAGGAGAGGAGAGGAGAGGAGAGGTTTCTTCTCTCTGGGTCTCGGATGAGTTTATTTACCAGCACGTG
CCGAATAAAAAGGCCTTTTTCTAAATGTGTTTTGATTGGTTGCAGATTTCTGAGCTGGTTGTGGAGGACA
GTGGTGTCTACTACTGCGCTGCTAGTCACGGTGAAACAACAGCCTGAAGAGCAGTACAAATACAA

>NC_068455.1- Oncket TRB1V6 - Chr35- Oket_V2 – 5176735-5180111

ATGACGCATCCTATAGTCCTCCTGAGTTGGCTGTTTCTCCTGTCCCCAAGTGAGTGTCTGAGGTTAGCTTTG
TAGCTGGGATTCTTGGAGTAATTAGAGTTGTACCAACTCTTAATAGACACAGTGATGGCTTGTCTCTCTTCT
TCCTCCTCTCTTCTCCTCTCTTCCTCTCCTGTCCCCTGATCCTCTCCCCTGCCCTCCTCTCCTCTCTTCTCTCTT
CTCAACAGGTCAGTGTGGAGCAGCAGGCCCCATAGTGCTTCAGCCCAGACAGCTCCTCACCCTTCCTGGT
AACACCACCACCTCCAGTCCAGTCACCCTGCAGTGCTCCCTGGGTCCAGGCTTCAGCATGCCCAGCTACA
CCATGATGTGGTACCGACAGGTCAGAGAAGTTACCAGGGCATTACACCCTGATGTGGTACTGATAGGTTA
GAGAAGTTATCAGGGCATTACACATGTCGGTACTCTCTTCTGTTGTCAGATCAGTGTTGGGCAACAGTGA
ACTGGATTTACTGCAACTAGTCATTTAATTACATTTTGCAATAGCTTGGTGGTAGTTGAACTAAATTAAAAT
CTTGGTAGTGTTTTCAATAGTTAATTACTGTTTTACCATGTAGCTAACTACTGGAACTACACACTACTGTTTT
ACCTGGTAGCGGTGTAGCTAACTACTGGAACTACACACTACTGTTTTACCATGTAGCTGTGTAGCTAACTAC
TGGAACTACACACTACTGTTTTTAGAGGTGTAGCTAACTACTGGAACTACACACTACTGTTTTACCATGTAG
CGGTGTAGCTAACTACTGGAACTACACACTACTGTTTTACCATGTAGCGGTGTAGCTAACTACTGGAACTA
CACACTACTGTTTTACCATGTAGCGGTGTAGCTAACTACTGGAACTACACACTACTGTTTTACCATGTAGCG
GTGTAGCTAACTACTGGAACTACACACTACTGTTTTACCATGTAGCGGTGTAGCTAACTACTGGAACTACAC
ACTACTGTTTTACCATGTAGCGGTGTAGCTAACTACTGGAACTACACACTACTGTTTTACCATGTAGCGGTG
TAGCTAACTACTGGAACTACACACTACTGCAGGTTGGTAGGTGGAGTGGGGTGGCCAGCAGGTTGGTAG
GTGAGGGGGGGTGGAGTGTCCAGCAGGTTGGTAGGTGGGGTGGGGTGGCCAGCAGGTTGGTAGGTG
AGGTGGGGTGGGGTGGCCAGCAGGTTGGTAGGTGAGGTGGGGTGAGGTGGAGTGGCCAGCAGGTTG
GTAGGTGAGGTGGGGTGGAGTGGCCAGCAGATTGGTAGGTGGGGTGGGGTGGGGTGGAGTGTCCAGC
AGGTTGGTAGGTGGGGTGGAGAGGCCAGCAGGTTGGTAGGTGAGGGGGTGGAGTGTCCAGCAGGTTG
GTAGGTGGGGTGGGGTGGCCAGCAGGTTGGTAGGTGAGGTGGGGTGGAGTGGGGTGGGGTGGCCAG
CAGGTTGGTAGGTGAGGTGGGGTGGAGAGGCCAGCAGATTGGTAGGTGAGGGGGGGTGGAGTGTCCA
GCAGGTTGGTAGGTGAGGTGGGGTGGAGAGGGGTGGCCAGCAGGTTGGTAGGTGAGGTGGGGTGGA
GAGGCCAGCAGATTGGTAGGTGAGGTGGGGTGGAGTGGGGTGGCCAGCAGGTTGGTAGGTGAGGTGG
GGTGGAGAGGCCAGCAGATTGGTAGGTGAGGGGGGTGGAGTGTCCAGCAGGTTGGTAGGTGGGGTG
GGGTGGAGTGGCCAGCAGATTGGTAGGTGAGGTGGGGTGGGGTGGAGTGGCCAGCAGATTCGTAGGT
GGGGTGGGGTGGAGAGGCCAGCAGGTTGGTAGGTGAGGTGGGGTGGAGTGGAGTGGGGTGGGGTG
GAGTGGCCAGCAGGTTGGTAGGTGAGGTGGGTTGGAGTGGAGTGGGGTGGGGTGGAGTGGCCAGCA
GGTTGGTAGGTGAGGTGGGGTGGGGTGGAGTGGCCAGCAGATTCATAGGTGGGGTGGGGTGGAGAG
GCCAGCAGGTTGGTAGGTGAGGTGAGGTGGGTTGGAGTGGCCAGCAGGTCTATAGGTGGGGTGGGGT
GGAGTGGCCAGCAGGTTGGTAGGTGAGGTGGGGTGGAGAGGCCAGCAGGTCTATAGGTGGGGTGGGG
TGGAGAGGCCAGCAGGTTGGTAGTTGGGGTGGGGTGGGGTGGAGTGGCCAGCAGGTTGGTAGTTGGG
GTGGGGTGGGGTGGAGTGGCCAGCAGGTTGGTAGTTGGGGTGGGGTGGGGTAGAGTGGCCAGCAGG
TTGGTAGGTGAGGTGGCCAGCAGATTCGTAAGTGGAGTGGGGTGGCCAGCAGGTTAGAAGGTGTGGTG
GCCAGCAGATTCATAGGTGGGGTGGGGTGGCCAGCAGGTTGGTAGGTGAGGTGGGGTGTCCAGCAGG
TTAGAAGGTGAGGTGGGGTGTCCAGCAGGTTGGTAGGTGAGGTGGGGTGTCCAGCAGGTTGGTAGGT
GAGGTGGGGTGTCCAGCAGGTTGGTAGGTGAGGTGGGGTGTCCAGCAGGTTGGTAGGTGTGGTGGGG
TGTCCAGCAGGTTATAAGGTGTGGTGGGGTGTCCAGCAGGTTAGAAGGTGAGGTGGGGTGGCCAGCAG
GTTAGAAGGTGAGGTGGGGTGTCCAGCAGGTTAGGTGTGGTACAATAACAAGCTTTGGTACCAACAGGT
CCGCTATGGAGGCCCAGTGGAGTTCCTCATTAAGGAGTACGAGAAAACCCAGGGGCGATACAAAGAGG
ACCTGGACACATCGGGGAACAGGTTCATCCTGCAGGTGATGGAGAGCATCTGAGCCGGGTTCCTCTTGA
CGTTTCTTCTCTCTGTGTAGAGGAGAGGAGAGGAGAGGAGAGGAGAGGAGAGGAGGAGAGGAGAGG
GAGAGGAGAGGAGAGGAGAGGAGAGGAGAGGAGAGGAGAGGAGAGTTTCTTCTCTCTGTGTAGAGA
GGAGGAGGAGGAGGAGGAGAGGAGAGGAGAGGAGGGGAGGAGGAGGAGAGAGAGAGGAGAGGA
GAGGAGAGGGAGAGGAGAGGAGAGGAGAGGAGAGGAGAGGAGAGGAGAGGAGAGGAGAGGAGA
GGAGAGGAGAGGAGAGGAGAGGAGAGGAGGAGGAGAGGTTTCTTCTCTCTGTGTAGAGAGGAGGAG

GAGGAGAGGAGAGAGGAGAGAGGAGAGGAGAGGAGAGGAGAGGAGAGGAGAGGAGAGGTTTCTT
CTCTCTGGGTCTCGGCTGAGTTTATTTACCAGCACGTGCCGAATAAAAAGGCCTTTTTCTAAATGTGTTTT
GATTGGTTGCAGATTTCTGAGCTGGTTGTGGAGGACAGTGGTGTCTACTACTGCGCTGCTAGTCACGGTG
AAACAACAGCCTGAAGAGCAGTACAAATACAA

>NC_060182.1-Oncgorb TRB1V6 - OgorEven_v1.0 - LG10 -95819849 - 95824099

ATGACGCATCCTATAGTCCTCTTGAGTTGGCTGTTTCTCCTGTCCCCAAGTGAGTGTCTGAGGTTCGCTTTG
TAGCTGGGATTCTTGGAGTAATTAGAGTTGTACCAACTCTTAAAAGACACAGTGATGGCTTGTCTCTCTTC
TTCCTCCTCTCTTCTCCTCTCTTCCTCTCCTGTCCCCTGATCCTCTTCCCTGCCCTCCTCTCCTCTCTTCTCAAC
AGGTCAGTGTGGAGCAGCAGGCCCCATAGTGCTTCAGCCCAGACAGTTCCTCACCCTTCCTGGTAACACC
ACCACCTCCAGTCCAGTCACCCTGCAGTGCTCCCTGGGTCCAGGCTTCAGCATGCCCAGCTACACCATGAT
GTGGTACCGACAGGTCAGAGAAGTTACCAGGGCATTACACCCTGATGTGGTACTGATAGGTTAGAGAAG
TTATCAGGGCATTACACCCTGATGTGGTACTGATAGGTTAGAGAAGTTACCAGGGCATTACACCATGATGT
GGTACTGATAGGTTAGAGAAGTTATCAGGGCATTACACCCTGATGTGGTACTGATAGGTCAGAAGTTATCA
GGGCATTACATCATGATGTGGTACTGATAGGTTAGAGAAGTTACCAGGGCATTACACCCTGATGTGGTACT
GATAGGTTAGAGAAGTTATCAGGGCATTACACCCTGATGTGGTACTGATAGGTTAGAGAAGTTACCAGGG
CATTACACCCTGATGTGGTACTGATAGGTTAGAGAAGTTACCAGGGCATTACACCCTGATGTGGTACTGAT
AGGTTAGAGAAGTTACCAGGGCATTACACCATGATGTGGTACTGATAGGTTAGAGAAGTTACCAGGGCAT
TACACCCTGATGTGGTACTGATAGGTTAGAGAAGTTACCAGGGCATTACACCCTGATGTGGTACTGATAGG
TTAGAGAAGTTACCAGGGCATTACACCCTGATGTGGTACTGATAGGTTAGAGAAGTTACCAGGGCATTAC
ACCCTGATGTGGTACTGATAGGTTAGAGAAGTTACCAGGGCATTACACCATGATGTGGTACTGATAGGTTA
GAGAAGTTACCAGGGCATTACACCATGATGTGGTACTGATAGGTTAGAGAAGTTACCAGGGCATTACACC
CTGATGTGGTACTGATAGGTTAGAGAAGTTACCAGGGCATTACACCCTGATGTGGTACTGATAGGTTAGAG
AAGTTACCAGGGCATTACACCCTGATGTGGTACTGATAGGTTAGAGAAGTTACCAGGGCATTACACCCTG
ATGTGGTACTGATAGGTTAGAGAAGTTACCAGGGCATTACACCCTGATGTGGTACTGATAGGTTAGAGAA
GTTACCAGGGCATTACACCATGATGTGGTACTGATAGGTTAGAGAAGTTACCAGGGCATTACACCCTGATG
TGGTACTGATAGGTTAGAGAAGTTATCAGGGCATTACACCCTGATGTGGTGCTGATAGGTTAGAGAAGTT
ATCAGGGCATTACACCCTGATGTGGTGCTGATAGGTTAGAGAAGTTATCAGGGCATTACACCCTGATGTGG
TACTGATAGGTCAGAAGTTACCAGGGCATTACACCATGATGTGGTACTGATAGGTCAGAAGTTACCAGGG
CATTACACCATGATGTGGTACTGATAGGTCAGAAGTTACCAGGGCATTACACCATGATGTGGTACTGATAG
GTCAGAAGTTACCAGGGCATTACACCATGATGTGGTACTGATAGGTCAGAAGTTACCAGGGCATTACACC
CTGATGTGGTACTGATAGGTTAGAGAAGTTACCAGGGCATTACATCATGATGTGGTACTGATAGGTTAGAG
AAGTTATCAGGGCATTACACCCTGATGTGGTGCTGATAGGTTAGAGAAGTTATCAGGGCATTACACCCTGA
TGTGGTGCTGATAGGTTAGAGAAGTTATCAGGGCATTACACCCTGATGTGGTACTGATAGGTTAGAGAAG
TTACCAGGGCATTACACATGTCGGTACTCTCTTCTGTTGTCGGATCAGTGTTGGGCGACAGTGAACTGGG
TTTACTGCAACTAGTCATTTAATTACATTTTGCAGTAGCTTGGTGGTAGTTGAACTAAATTAAAATCTTGGTA
GTGTTTTCAGTAGTTAATTACTGTTTTACCATGTAGCTAACTACTGGAACTACACACTACTGTTTTACCATGT
AGCAGTGTAGCTAACTACTGGAACTACACACTACTGTTTTACCATGTAGAGGTGTAGCTAACTACTGGAAC
TACGCGTCCGCTAAATGGCATATATTCTACGTATGGGTGGTCCCGGGAATCGAACCCACTACCCTGGCGTT
ACAAGCGCCATGCTCTACCAACTGAGCGGTGTAGCTAACTACTGGAACTACACACTACTGTTTTACCATGT
AGCGGTGTAGCTAACTACTGGGACTATACACTACTGTTTTACCATGTAGCGGTGTAGCTAACTACTGGAAC
TACACACTACTGTTTTACCATGTAACAGTGTAGCTAACTACTGGAACTACACACTACTGTTTTTAGAGGTGT
AGCTAACTACTGGAAATACACACTACTGTTTTACCATGTAGCGGTGTAGCTAACTACTGGAACTGCACACT
ACTGTTTTACCATGTAGCGGTGTAGCTAACTACTGGAACTACACACTACTGCAGGTTGGTTGGTGGAGTG
GGGTGGCCAGCAGGTTGGTAGGTGAGGGGGGGTGGAGTTTCCAGCAGGTTGGTAGGTGGGGTGGGG

TGGCCAGCAGGTTGGTAGGTGAGGTGGGGTGGAGTGGGGTGGGGTGGGGTGGCCAGCAGGTCTATAG
GTGGGGTGGGGTGGAGAGGCCAGCAGGTTGGTAGGTGGGGTGGGGTGGCCAGCAGGTTGGTAGGTG
AGGTGGGGTGGAGTGGGGTGGGGTGGAGTGGCCAGCAGATTGGTAGGTGGGGTGGGGTGGAGAGGC
CAGCAGGTTGGTAGGTGGGGTGGGGTGGCCAGCAGGTTGGTAGGTGAGGTGGGGTGGAGTGGGGTG
GGGTGGAGTGGCCAGCAGATTGGTAGGTGGGGTGGGGTGGAGAGGCCAGCAGATTGGTAGGTGGGGT
GGGGTGGAGAGGCCAGCAGGTTGGTAGTTGGGGTGGGGTGGGGTAGAGTGGCCAGCAGGTTGGTAG
GTGAGGTGGGGTGGGGTGGCCAGCAGGTTAGAAGGTGTGGTGGGGTGTCCAGCAGGTTAGAAGGTGT
GGTGGGGTGGCCAGCAGGTTGGTAGGTGAGGTGGGGTGTCCAGCAGGTTATAAGGTGTGGTGGGGTG
TCCAGCAGGTTAGAAGGTGTGGTGGGGTGTCCAGCAGGTTAGAAGGTGTGGTGGGGTGGCCAGCAGG
TTGGTAGGTGAGGTGGGGTGGCCAGCAGGTTGGCCAGCAGGTTGGTAGGTGGTCCTTCTGTAGCTCAG
TTGGTAGAGCATGGCCCTTGTAACGCCAGGGTAGTGGGTTCGATTCCCGGGATCACCCATACGTAGAATG
TATGCACACATGACTGTAAGTCGCTTTGGATAAAAGCGTCTGCTAAATGGCATATATTATTATTATATATTAG
GTTGGCAGGTGAGGTGGGGTGGCCAGCAGGTTATAAGGTGAGGTGGGGTGTCCAGCAGGTTGGAAGG
TGAGGTGGGGTGGCCAGCAGGTTGGAAGGTGAGGTGGGGTGTCCAGCAGGTTAGAAGGTGAGGTGG
GGTGGCCAGCAGGTTAGAAGGTGAGGTACAATAACAAGCTTTGGTACCAACAGGTCCGCTATGGAGGCC
CAGTGGAGTTCCTCATTAAGGAGTACGAGAAAACCCGGGGGCGATACAAAGAGGACCTGGACACATCG
GGGAACAGGTTCATCCTGCAGGTGATGGAGAGCATCTGAGCCGGGTTCCTCTTGACGTTTCTTCTCTCTG
TGTAGAGGAGAGGAGAGGAGAGGAGAGGAGAGGGAGAGGAGAGGAGAGGAGAGGAGAGGGAGAG
GAGAGGAGAGGAGAGGAGGAGAGGAGAGGAGAGGAGAGGAGAGGAGAGGAGAGGAGAGGAGAG
GAGAGGAGAGGAGAGGAGAGGAGAGGAGAGGAGAGGTTTCTTCTCTCTGGGTCTCGGCTGAGTTTAT
TTACCAGCACATGCCGAATAAAAAGGCCTTTTTCTAAATGTGTTTTGATTGGTTGCAGATGTCTGAGCTGG
TTGTGGAGGACAGTGGTGTCTACTACTGCGCTGCTAGTCACGGTGAAACAACAGCCTGAAGAGCAGTAC
AAATACAA
