# Supplementary material for: Evolution of T cell receptor beta loci in salmonids
Source: Front Immunol. 2023 Aug 15;14:1238321. doi: 10.3389/fimmu.2023.1238321 (PMC10464911; doi:10.3389/fimmu.2023.1238321)
Supplement: Supplementary file 4 [file DataSheet_4.pdf]

Supplementary File 4.

RPKM values in various salmonid transcriptomes that support the expression of the different TRBC subgroups.

RPKM values in various salmonid transcriptomes

Salmo salar	TRB01 (394 bp)	TRB09 (510 bp)	SRR	# reads
HK	70,79	248,16	SRR1422860	59.084.708
Kidney	22,49	65,71	SRR1422864	61.054.936
Gills	20,63	58,50	SRR1422858	59.793.962
Spleen	55,19	248,15	SRR1422870	60.203.316
Gut	18,16	44,82	SRR1422859	59.806.348
Nose	5,84	21,44	SRR1422867	59.545.012
Heart	1,00	3,34	SRR1422862	58.163.180
Brain	0,43	1,43	SRR1422856	58.939.250
Liver	1,60	5,47	SRR1422865	58.784.272
Salmo trutta	Chr.33 (510 bp)	Chr.25 (567 bp)		
Juvenile mixed tissue	2,73	6,72	SRR6666113	93.920.774
Oncorhynchus kisutch	Chr.7 (486 bp)	Chr.14 (510 bp)		
Spleen	18,25	84,04	SRR5333360	133.685.908

RPKM values shown in Table 2 were defined using CLC Genomic Workbench 6.0.5 (CLC Genomics Workbench 6. https://www.qiagenbioinformatics.com/products/clc-genomics-workbench). Read mapping was performed with the stringency of 98% identity and 95% coverage. Nucleotide sequence lengths used in the analysis is indicated in parenthesis in base pairs (bp) where only open reading frame TRBC sequences were used. Chromosome location of transcripts matching the Atlantic salmon TRB01 and TRB09 sequences originate from the NCBI *Oncorhynchus kisutch* GCA_002021735.2 and *Salmo trutta* GCA_901001165.1 genomes.
