# Supplementary material for: Evolution of T cell receptor beta loci in salmonids
Source: Front Immunol. 2023 Aug 15;14:1238321. doi: 10.3389/fimmu.2023.1238321 (PMC10464911; doi:10.3389/fimmu.2023.1238321)
Supplement: Supplementary file 5 [file DataSheet_5.pdf]

Supplementary File 5. ClustalW alignment of deduced amino acid sequences from TRBC genes annotated in salmonid genomes.

The domains and regions are indicated based on IMGT numbering: C(constant domain), CO (connecting region), TM (transmembrane region) and CY (cytoplasmic region).

We use TRBC sequences identified in Salmonid genome assemblies, as well as human TRBC1 (P01850.4) and TRBC2 (A0A5B9.2) gene amino acid sequences. Specifically, rainbow trout (*Oncorhynchus mykiss*), Arlee and Swanson lines: GCA_013265735.3 (NC_048589.1: from 43176761 to 43177117, from 43979157 to 43979516; NC_048583.1: from 63763500 to 63763853) and GCA_025558465.1(CM046594.1: from 92821775 to 92822134, from 88287356 to 88287715; CM046588.1: from 59036934 to 59037287), Atlantic salmon (*Salmo salar*): GCA_905237065.2 (NC_059450.1: from 48494763 to 48495119, from 47732112 to 47732468; NC_059442.1: from 3712267 to 3712623, from 4431095 to 4431451), brown trout (*Salmo trutta*): GCA_901001165.1 (NC_042981.1: from 3820220 to 3820576, from 3360038 to 3360394; NC_042989.1: from 41909413 to 41909754; from 41146330 to 41146671), Coho salmon (*Oncorhynchus kisutch*): GCA_002021735.2 (NC_034187: from 4131571 to 4131924; from 4419372 to 4419725; NC_034180.2: from 54067269 to 54067577; from 54585344 to 54585652) , Chinook salmon (*Oncorhynchus tshawytscha*): GCA_002872995.1 (NC_037104.1: from 73073875 to 73074138, from 73058750 to 73059103, from 73048533 to 73048739;), Pink salmon (*Oncorhynchus gorbuscha*) (even and odd year): GCA_021184085.1 (NC_060182.1: from 95791252 to 95791605, from 96054336 to 96054689; NC_060189.1: from 1592848 to 1593186, from 2463629 to 2463967) and GCA_017355495.1 (CM029856.1: from 93543873 to 93544226, from 93300858 to 93301136; CM029863.1: from 1668337 to 1668675, from 2284373 to 2284711), and Northern pike (*Esox lucius*): GCA_004634155.1(NC_025982.4: from 7137435 to 7137779, from 6848109 to 6848413).

Esoluc: *Esox Lucius;* Oncmyk: *Oncorhynchus mykiss*; Salsal: *Salmo salar;* Onctsh: *Oncorhynchus tshawytscha;* Onckis: *Oncorhynchus kisutch*, Oncgor: *Oncorhynchus gorbuscha; Homsap: Homo sapiens*.

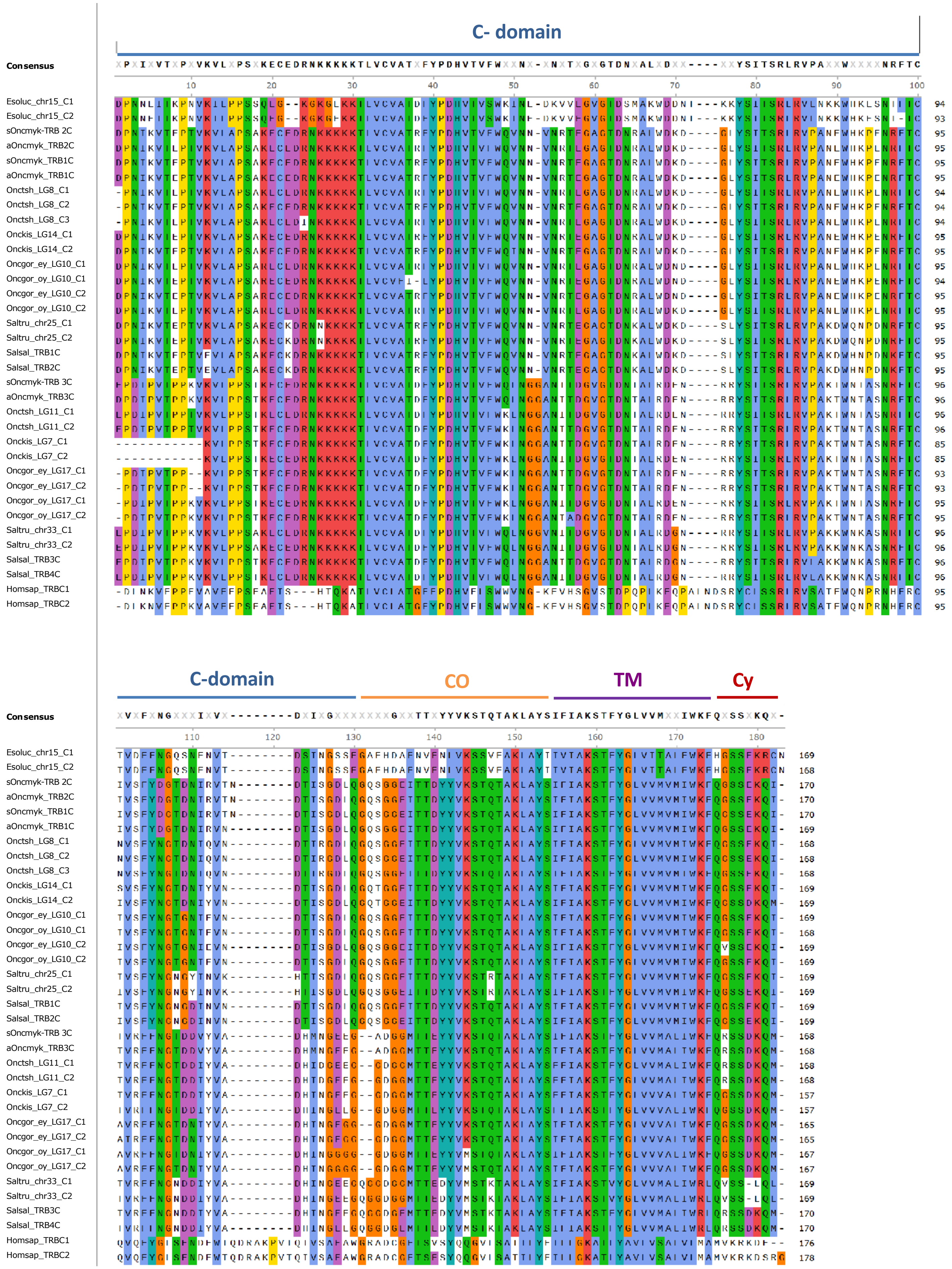

C- domain
Consensus
Esoluc_chr15_C1
Esoluc_chr15_C2
sOncmyk-TRB 2C
aOncmyk_TRB2C
sOncmyk_TRB1C
aOncmyk_TRB1C
Onctsh_LG8_C1
Onctsh_LG8_C2
Onctsh_LG8_C3
Onckis_LG14_C1
Onckis_LG14_C2
Oncgor_ey_LG10_C1
Oncgor_oy_LG10_C1
Oncgor_ey_LG10_C2
Oncgor_oy_LG10_C2
Saltru_chr25_C1
Saltru_chr25_C2
Salsal_TRB1C
Salsal_TRB2C
sOncmyk-TRB 3C
aOncmyk_TRB3C
Onctsh_LG11_C1
Onctsh_LG11_C2
Onckis_LG7_C1
Onckis_LG7_C2
Oncgor_ey_LG17_C1
Oncgor_ey_LG17_C2
Oncgor_oy_LG17_C1
Oncgor_oy_LG17_C2
Saltru_chr33_C1
Saltru_chr33_C2
Salsal_TRB3C
Salsal_TRB4C
Homsap_TRBC1
Homsap_TRBC2
C-domain
CO
TM
Cy
